# Inflammation Fuels Colicin Ib-Dependent Competition of *Salmonella* Serovar Typhimurium and *E. coli* in *Enterobacterial* Blooms

**DOI:** 10.1371/journal.ppat.1003844

**Published:** 2014-01-02

**Authors:** Lubov Petkova Nedialkova, Rémy Denzler, Martin B. Koeppel, Manuel Diehl, Diana Ring, Thorsten Wille, Roman G. Gerlach, Bärbel Stecher

**Affiliations:** 1 Max-von-Pettenkofer Institute, LMU Munich, Munich, Germany; 2 German Center for Infection Research (DZIF), partner site LMU Munich, Munich, Germany; 3 Institute of Microbiology, ETH Zürich, Zürich, Switzerland; 4 Robert Koch-Institut, Wernigerode Branch, Junior Research Group 3, Wernigerode, Germany; Yale University School of Medicine, United States of America

## Abstract

The host's immune system plays a key role in modulating growth of pathogens and the intestinal microbiota in the gut. In particular, inflammatory bowel disorders and pathogen infections induce shifts of the resident commensal microbiota which can result in overgrowth of *Enterobacteriaceae* (“inflammation-inflicted blooms”). Here, we investigated competition of the human pathogenic *Salmonella enterica* serovar Typhimurium strain SL1344 (*S.* Tm) and commensal *E. coli* in inflammation-inflicted blooms. *S.* Tm produces colicin Ib (ColIb), which is a narrow-spectrum protein toxin active against related *Enterobacteriaceae*. Production of ColIb conferred a competitive advantage to *S.* Tm over sensitive *E. coli* strains in the inflamed gut. In contrast, an avirulent *S.* Tm mutant strain defective in triggering gut inflammation did not benefit from ColIb. Expression of ColIb (*cib*) is regulated by iron limitation and the SOS response. CirA, the cognate outer membrane receptor of ColIb on colicin-sensitive *E. coli*, is induced upon iron limitation. We demonstrate that growth in inflammation-induced blooms favours expression of both *S.* Tm ColIb and the receptor CirA, thereby fuelling ColIb dependent competition of *S.* Tm and commensal *E. coli* in the gut. In conclusion, this study uncovers a so-far unappreciated role of inflammation-inflicted blooms as an environment favouring ColIb-dependent competition of pathogenic and commensal representatives of the *Enterobacteriaceae* family.

## Author Summary

Colicins are bacterial protein toxins which show potent activity against sensitive strains *in vitro*. Ecological models suggest that colicins play a major role in modulating dynamics of bacterial populations in the gut. However, previous studies could not readily confirm these predictions by respective *in vivo* experiments. In animal models, colicin-producing strains only show a minor or even absent fitness benefit over sensitive competitors. Here, we propose that the gut environment plays a crucial role in generating conditions for bacterial competition by colicin Ib (ColIb). Gut inflammation favours overgrowth of *Enterobacteriaceae* (“inflammation-inflicted *Enterobacterial* blooms”). We show that a pathogenic *Salmonella* Typhimurium (*S.* Tm) strain benefits from ColIb production in competition against commensal *E. coli* upon growth in inflammation-inflicted blooms. In the absence of gut inflammation, ColIb production did not confer a competitive advantage to *S.* Tm. In the inflamed gut, the genes for ColIb production in *S.* Tm and its corresponding ColIb-surface receptor CirA in *E. coli* were markedly induced, as compared to the non-inflamed gut. Therefore, environmental conditions in inflammation-inflicted blooms favour colicin-dependent competition of *Enterobacteriaceae* by triggering ColIb production and susceptibility at the same time. Our findings reveal a role of colicins as important bacterial fitness factors in inflammation-induced blooms.

## Introduction

Enteric *Salmonella enterica* serovar Typhimurium (*S.* Tm) infection is antagonized by a highly complex intestinal microbiota, a condition termed colonization resistance. Disruption of colonization resistance by oral antibiotic therapy, germfree state or an immature microbiota of low complexity results in increased susceptibility to oral infection with pathogens of the *Enterobacteriaceae* family [1], [2], [3]. In addition to the named conditions, inflammatory changes in the intestine induce dysbiosis and favour *Enterobacteriaceae* overgrowth [4], [5], [6]. Recently, we have shown that *S.* Tm-induced gut inflammation mediates parallel blooms of *S.* Tm and host-intrinsic commensal *E. coli* [7], [8]. In these blooms, both bacteria can reach high densities (>10^8^ cfu/ml) while the residual microbiota is outgrown [7]. Therefore, under inflammatory conditions, commensal *E. coli* are one of the main competitors of *S.* Tm. In the inflamed gut, environmental conditions encountered by bacteria vastly differ from the situation in the absence of inflammation. By resisting antimicrobial defences, utilizing iron acquisition systems and exploiting anaerobic electron acceptors (e.g. NO_3_^−^, tetrathionate), *Enterobacteriaceae* can capitalize on inflammatory conditions [9], [10], [11], [12], [13], [14].

Besides exploitative competition for resources, bacteria can directly antagonize one another by producing antimicrobials, such as bacteriocins. Bacteriocins produced by *Enterobacteriaceae* (*E. coli*, *Salmonella* and relatives) are termed colicins. They have a narrow spectrum of activity and act only against phylogenetically close relatives. Colicins kill by one of three general mechanisms: pore formation in the inner membrane, nuclease activity or interference with cell wall synthesis [15]. We have shown that growth of *S.* Tm in inflammation-induced blooms promotes horizontal transfer of the conjugative pCol1B9-plasmid (in the following termed P2) to commensal *E. coli* strains [7]. P2 encodes the locus for colicin Ib (ColIb) production (*cib*) and immunity (*imm*). In blooms, ColIb was shown to increase the fitness of *S.* Tm in competition with commensal, colicin-sensitive *E. coli*. However, it has remained unclear if ColIb only affords a benefit for *S.* Tm in inflammation-inflicted blooms or also in the absence of gut inflammation.

ColIb belongs to the group of pore-forming colicins [16]. ColIb is closely related to ColIa and most of its structural/functional properties can be inferred from ColIa [17]. Free ColIa/b binds to the outer-membrane receptor CirA, a catecholate siderophore receptor, and traverses the outer membrane in a TonB-dependent way [18], [19], [20]. The exact mode of outer-membrane translocation is still unclear but a recent study suggests that two molecules of CirA are required, one for ColIa/ColIb binding, the second one for its translocation [18], [21]. ColIa/ColIb forms a pore in the inner membrane of sensitive bacteria which leads to disruption of the electrochemical membrane gradient and consequent bacterial death [22]. ColIa/ColIb producers protect themselves by expression of the immunity protein (*imm*) which interferes with ColIa/ColIb action in the inner membrane [23]. In addition, resistance to ColIb can be gained by alterations of the cell-surface receptor CirA or mutations in the TonB-dependent import pathway [24].

ColIa/ColIb expression is regulated in a Fur- and LexA-dependent way [25]. The Fur protein binds Fe^II^ and thereby measures the intracellular Fe^II^ pool [26]. In the iron-bound state, Fur acts as a transcriptional repressor of iron-regulated promoters which is released under iron-limiting conditions. In addition, the ColIb gene *cib* is also repressed by the LexA protein, the regulator of the SOS response. The SOS response is launched when bacteria sense DNA damage (e.g. DNA double strands breaks) [27]. As a consequence, the RecA protease is activated and cleaves the LexA repressor protein which in turn activates an array of genes involved in DNA repair, survival, prophages and also colicins [15], [28]. Interestingly, ColIa and ColIb are the only reported colicins which are controlled by both, Fur and LexA. This suggests that maximal expression of ColIa/b requires iron limitation and stress conditions. Interestingly, the majority of other colicins are only regulated by LexA and usually contain two LexA-binding sites in their promoter to ensure tight repression.

Theoretical and experimental studies suggested that colicin producers have a competitive advantage over non-producers when colonizing the same ecological niche [29]. When directly tested in competition experiments with sensitive strains in animal models, colicin production only conferred a competitive advantage after weeks [30], [31]. In some cases, colicin-production even conferred no detectable benefit despite colicin-dependent killing could be demonstrated under *in vitro* conditions [32], [33], [34]. The underlying reasons were attributed to colicin inactivation by proteases [33] or reduced colicin activity under anaerobic conditions [35], [36].

In the *S.* Tm mouse colitis model ColIb conferred an overt fitness benefit for *S.* Tm in competition against a colicin-sensitive *E. coli* strain [7]. This was somewhat surprising, considering the lack of an apparent competitive advantage of colicin producers in the reports mentioned above. Although different experimental setups were used in previous studies, a key difference to our study was the lack of concomitant gut inflammation. Therefore we reasoned that the inflammatory response may somehow promote ColIb dependent competition of *S.* Tm and *E. coli*. We tested this idea and analyzed ColIb-dependent *S.* Tm and *E. coli* competition under normal and inflammatory conditions in the mouse colitis model. Our experiments revealed that the inflammatory response creates conditions that potentiate the effects of colicins, both by increasing their production and by mediating susceptibility of the competitor. This finding has implications for colicin ecology and points out the importance of colicins as fitness factor for bacterial competition in *Enterobacterial* blooms in the inflamed gut.

## Results

### ColIb affords *S.* Tm a growth advantage over colicin-sensitive *E. coli* strains in the inflamed, but not in the normal gut

**Figure 1:**
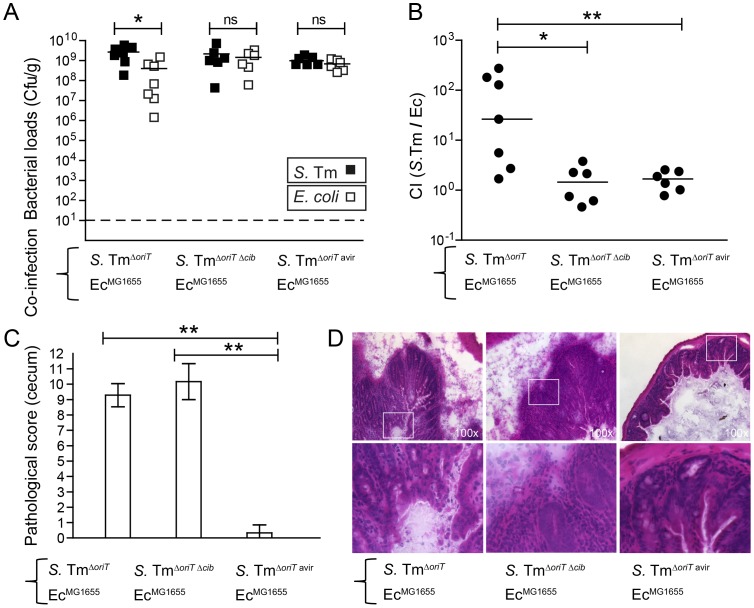
Colicin-dependent competition of *S.* Tm and *E. coli* in the gut in inflammation-induced “blooms” in gnotobiotic LCM mice. Streptomycin-treated LCM-mice were co-infected with 1∶1 mixtures of *S.* Tm^Δ*oriT*^ and Ec^MG1655^, *S.* Tm*^oriT^* ^Δ*cib*^ and Ec^MG1655^ or *S.* Tm^Δ*oriT* avir^ and Ec^MG1655^. (**A**) *S.* Tm (black) and *E. coli* (white) colonization densities were determined at day 4 p.i. in the cecum content (cfu/g). (**B**) Competitive indices (CI; ratio of *S.* Tm/*E. coli*) were determined for individual mice shown in (A). Bars show the median. (**C**) Histopathological analysis of cecal tissue of the infected mice shown in (A). Cecal tissue sections of the mice were stained with hematoxylin/eosin (H&E) and the degree of submucosal edema, neutrophil infiltration, epithelial damage and loss of goblet cells was scored (Materials and Methods). 1–3: no pathological changes; 4–7: moderate inflammation; above 8: severe inflammation. shown mean and StD. (**D**) Representative H&E–stained cecal sections of mice shown in (**A–C**). Magnification 100-fold. Enlarged sections (squares) are shown in the lower panels. Dotted line: detection limit (1 cfu/g).

ColIb confers a fitness benefit to *Salmonella* Typhimurium (*S.* Tm) over colicin-sensitive *E. coli* strains [7]. *In vitro*, the *E. coli* Nissle (Ec^Nissle^) strain shows intermediate susceptibility to ColIb (turbid inhibition zone), while the K-12 strain *E. coli* MG1655 (Ec^MG1655^) is highly sensitive (clear inhibition zone; Figure S1A). For this reason we selected Ec^MG1655^ for our initial experiments. First we tested, if the growth benefit of *S.* Tm over Ec^MG1655^ is ColIb-dependent. We performed the co-infection experiments with *S.* Tm strains and Ec^MG1655^ in the streptomycin *Salmonella* mouse colitis model [2]. Here, we used gnotobiotic mice colonized with a low-complexity microbiota (LCM) lacking any kind of *Enterobacteriaceae* which may interfere with the experiment (i.e. by the production of other colicins). Further, since the *S.* Tm P2-plasmid is highly mobile and rapidly transferred to co-colonizing *E. coli* strains in the gut [7], all *S.* Tm strains carried an additional mutation in the origin of transfer of P2 (Δ*oriT*) to prevent conjugation (Table 1). LCM mice pre-treated with streptomycin were co-infected with 1∶1 mixtures of Ec^MG1655^ and either ColIb-producing (*S.* Tm^Δ*oriT*^) or ColIb-deficient strains (*S.* Tm^Δ*oriT* Δ*cib*^). Ec^MG1655^ was efficiently outcompeted by *S.* Tm^Δ*oriT*^ but not by the ColIb-deficient mutant (Figure 1A,B). Both *S.* Tm strains induced similar degrees of gut inflammation by day 4 p.i. (Figure 1CD). This confirms that the competitive advantage of *S.* Tm over Ec^MG1655^ in the inflamed intestine is for the most part ColIb-dependent. Next, we tested if an avirulent strain (*S.* Tm^Δ*oriT* avir^) defective in triggering an inflammatory response due to the absence of functional type three secretion systems [37] would also benefit from ColIb. Interestingly, in the absence of gut inflammation, *S.* Tm^Δ*oriT* avir^ did not out-compete Ec^MG1655^ (Figure 1).

**Table 1:** Bacteria and plasmids used in this study.

*S.*Tm strains	*Lab-internal strain number*	*Genotype*	*Reference*
*S.* Tm^wt^	SB300	*S.* Tm strain SL1344	[77]
*S.* Tm^Δ*cib*^	M990	p2 *cib imm*::*aphT*	[7]
*S.* Tm^wt amp^	SB300	*S.* Tm carrying plasmid pWKS30 [71]	[7]
*S.* Tm^Δ*oriT*^	M1407	p2 Δ*oriT nikA*::*cat*	This study
*S.* Tm^avir^ p2^cm^	M996	Δ*invG*; *sseD::aphT* p2::*cat*	[7]
*S.* Tm^Δ*oriT* Δ*cib*^	MAD1	p2 Δ*oriT nikA*::*cat cib imm*::*aphT*	[7]
*S.* Tm^ΔP2 avir^	MBK15	Δ*invG*; *sseD::aphT* cured of pColIB plasmid (P2)	This study
*S.* Tm^avir^	M557	Δ*invG*; *sseD::aphT*	[37]
*S.* Tm^Δ*oriT* avir^	LPN5	Δ*invG*; *sseD::aphT* [37]; p2Δ*oriT nikA*::*cat*	This study
***E. coli*** ** strains**			
Ec DH5α			Invitrogen
Ec BL21 DE3			Stratagene
Ec^Nissle^			[7]
Ec^8178^			[7]
Ec^MG1655^		*E. coli* K-12 wild type strain MG1655, streptomycin-resistant	[78]
Ec^MG1655 Δ*cirA*^	LPN2	*cirA*::*aphT*	This study
**Plasmids**			
	pWKS29 and pWKS30		[71]
	pM979	Constitutive *gfp*mut2-reporter plasmid (ribosomal *rpsM* promoter)	[69]
	pLB02	Firefly-luciferase reporter plasmid	[70]
	pC831-2	expression of the ColIb (*cib*) immunity protein gene *imm*	[7]
	pLPN13	*cirA-6-x-his*	This study
	pLPN14	*cib-6-x-his*	This study
p*^cirA^* ^-*luc*^	pLPN15	*cirA*-promoter firefly-luciferase reporter	This study
p*^cib^* ^-*luc*^	pLPN16	*cib*-promoter firefly-luciferase reporter	This study
	pM1437	*cib*-promoter gfp-reporter	This study
	pLPN1	*cirA*-promoter gfp-reporter	This study
p^compl. *cirA*^	pWRG693-1	*cirA* complementation vector	This study
p^compl. *cib*^	pWRG694	*cib* complementation vector	This study

**Figure 2:**
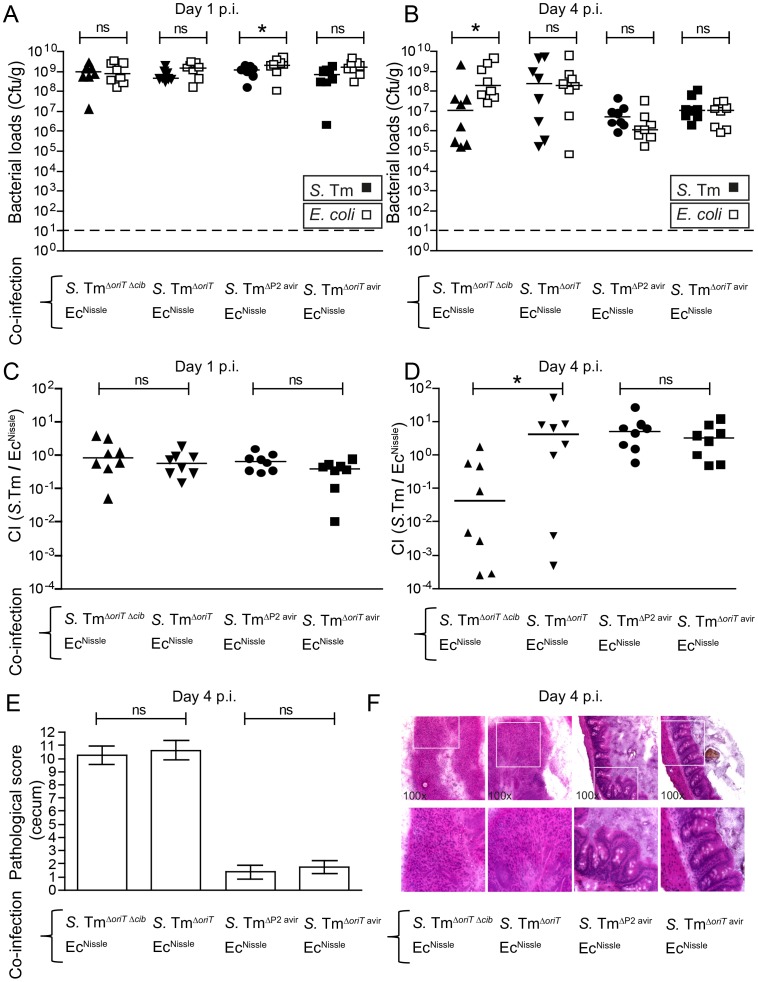
Colicin-dependent competition of *S.* Tm and *E. coli* in the gut in inflammation-induced “blooms” in conventional mice. Streptomycin-treated conventional mice were co-infected with 1∶1 mixtures of *S.* Tm^Δ*oriT* Δ*cib*^ and Ec^Nissle^, *S.* Tm^Δ*oriT*^ and Ec^Nissle^, *S.* Tm^ΔP2 avir^ and Ec^Nissle^ or *S.* Tm^Δ*oriT* avir^ and Ec^Nissle^. *S.* Tm (black) and *E. coli* (white) colonization densities (Cfu/g) were determined at day 1 (**A**) and day 4 p.i. (**B**) in the feces and cecum content respectively. (**C, D**) Competitive indices (CI; ratio of *S.* Tm/*E. coli*) were determined for individual mice shown in (A) and (B). Bars show the median. (**E**) Histopathological analysis of cecal tissue of the infected mice. Cecal tissue sections of the mice were stained with hematoxylin/eosin (H&E) and the degree of submucosal edema, neutrophil infiltration, epithelial damage and loss of goblet cells was scored (Materials and Methods). 1–3: no pathological changes; 4–7: moderate inflammation; above 8: severe inflammation. Bars show mean and StD. (**F**) Representative H&E–stained cecal sections of mice shown in (A–E). Magnification 100-fold. Enlarged sections (squares) are shown in the lower panels. Dotted line: detection limit (1 cfu/g).

Previous studies on colicin-dependent bacterial competition were using conventional mice. To this end we aimed to verify that our key findings in gnotobiotic mice were also reproducible in a more “natural” mouse model. To this end, we used the streptomycin-treated mice with a conventional complex microbiota. We used Ec^Nissle^ for the co-infection experiments as in contrast to LCM mice, Ec^MG1655^ only poorly colonized conventional streptomycin-treated, *S.* Tm^Δ*oriT*^ infected mice (not shown). We set up four different experimental groups of streptomycin-treated mice (Figure 2). One group was co-infected with a virulent colicin-producing, the other with a virulent colicin–deficient *S.* Tm strain (*S.* Tm^Δ*oriT*^ or *S.* Tm^Δ*oriT* Δ*cib*^, respectively) and Ec^Nissle^. Both groups developed strong *Salmonella*-induced gut inflammation upon infection by day 4 p.i. (Figure 2E,F). The other two groups were either co-infected with avirulent ColIb-producing *S.* Tm (*S.* Tm^Δ*oriT* avir^) and Ec^Nissle^, or avirulent ColIb-deficient *S.* Tm (*S.* Tm^ΔP2 avir^) and Ec^Nissle^. The latter two groups did not develop gut inflammation within 4 days of infection (Figure 2E,F). In the presence of inflammation, virulent ColIb-producing *S.* Tm^Δ*oriT*^ grew up to similar numbers as Ec^Nissle^ (to ∼10^8^ cfu/g) while ColIb-deficient *S.* Tm^Δ*oriT* Δ*cib*^ was outcompeted by Ec^Nissle^ (Figure 2B). This difference is also reflected in the competitive index (Figure 2D). In the absence of inflammation, both co-infecting strains (ColIb-producing *S.* Tm^Δ*oriT* avir^ and Ec^Nissle^) and (ColIb-deficient *S.* Tm^ΔP2 avir^ and Ec^Nissle^) colonized well at day 1 post infection (Figure 2AC) but were out-competed (to ∼10^7^ cfu/g) by the complex conventional microbiota, which recovers by day 5 after streptomycin-treatment (Figure 2B). No benefit of ColIb-production was observed for the avirulent *Salmonella* strain. The absolute ratio of *Salmonella*/Ec^Nissle^ is different when compared to *Salmonella*/Ec^MG1655^ in LCM mice (Figure 1). We reason that this is due to strain-specific differences between Ec^MG1655^ and Ec^Nissle^ as well as due to differences between the gnotobiotic and complex gut microbiota. This data verified that *S.* Tm/Ec^Nissle^ competition is only ColIb-dependent in the presence of gut inflammation.

In conclusion, these experiments prompted us to hypothesize that, in the normal non-inflamed gut, either ColIb expression by *S.* Tm was down-regulated or susceptibility of the Ec strains to ColIb was decreased. To further address the mechanism of colicin-dependent competition in the inflammation-induced blooms we investigated the regulation of *cib* expression in *S.* Tm as well as the susceptibility of Ec to ColIb in detail.

### Regulation of *S.* Tm ColIb is induced by iron limitation and the SOS response *in vitro*

**Figure 3:**
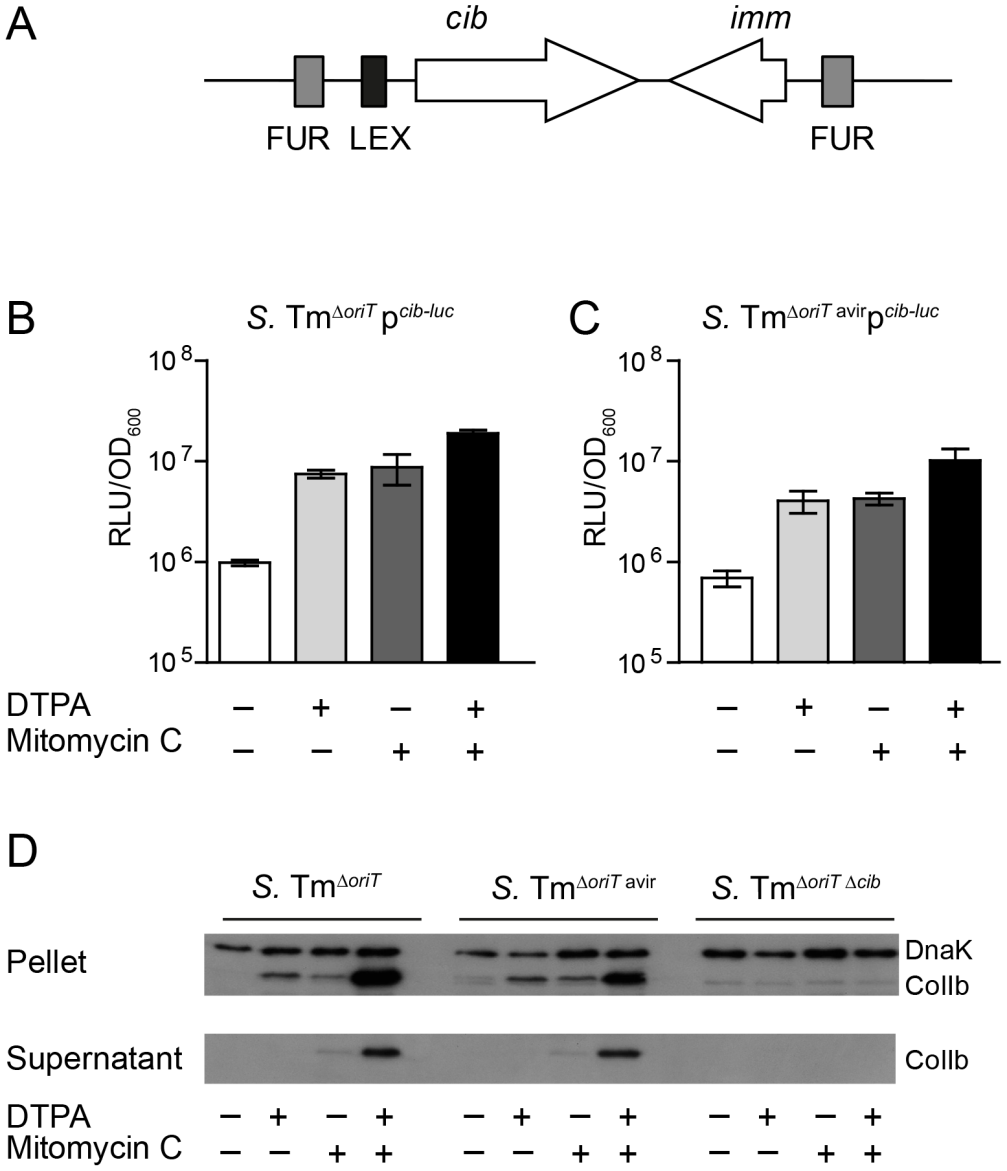
Expression of *S.* Tm ColIb is induced by iron limitation and the SOS response. (**A**) Organization of the ColIb locus showing location of the Fur and LexA repressor binding sites in the ColIb (*cib*) promoter and the immunity protein gene (*imm*). Overnight cultures of *S.* Tm^Δ*oriT*^ (**B**) and *S.* Tm^Δ*oriT* avir^ (**C**) carrying the reporter plasmid p*^cib^* ^-*luc*^ were re-inoculated 1∶20 in fresh LB with indicated supplements (0.25 µg/ml mitomycin C; 100 mM DTPA) and grown under aeration for 4 h. Cultures were normalized to OD_600_, bacteria were harvested and luciferase-activity was measured in bacterial lysates. Relative luminescence units (RLU) per unit OD_600_ are indicated. (**D**) Overnight cultures of indicated *S.* Tm strains were re-inoculated 1∶20 in fresh LB with indicated supplements (0.25 µg/ml mitomycin C; 100 mM DTPA) and grown under aeration for 4 h. OD_600_ of the cultures was normalized, bacteria were harvested and ColIb was detected in bacterial lysates as well as in the culture supernatant by immunoblot using an affinity-purified rabbit-α-ColIb antiserum. *S.* Tm DnaK was detected as loading control.

The *cib* promoter region contains binding sites for the transcriptional repressors Fur and LexA (Figure 3A; Figure S2). We generated a *cib* promoter *firefly-luciferase* (*luc*)-reporter construct as well as an affinity-purified polyclonal rabbit-α-ColIb antiserum to analyze the regulation of ColIb expression. ColIb expression was strongly up-regulated upon induction of the SOS response by the antibiotic mitomycin C (0.25 µg/ml) as confirmed by *luc*-reporter assays and immunoblot (Figure 3B–D). Depletion of Fe^(III)^ from culture media by chelation with 100 µM diethylenetriaminepentaacetic acid (DTPA) [38] had a comparable inductive effect on ColIb production. Supplementation of both, mitomycin C and DTPA lead to maximal induction of ColIb production and secretion. This result confirmed that ColIb is de-repressed in response to SOS signals and iron starvation.

### Upregulation of Ec^MG1655^ *cirA* under iron limitation correlates with increased ColIb susceptibility

**Figure 4:**
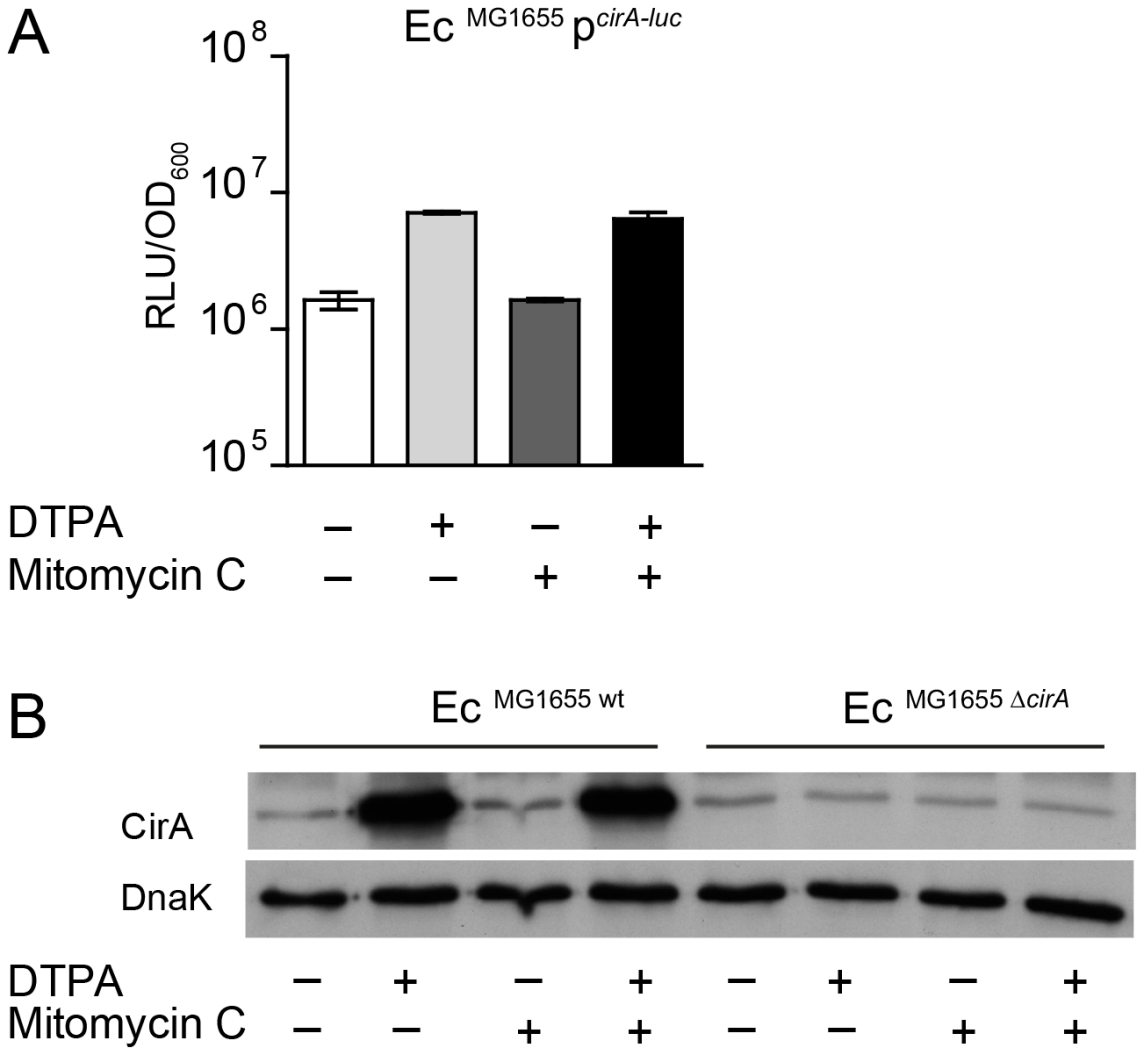
*E. coli cirA* expression is upregulated in response to iron limitation. (**A**) An overnight culture of Ec^MG1655^ carrying the reporter plasmid p*^cirA^* ^-*luc*^ was re-inoculated 1∶20 in fresh LB with indicated supplements (0.25 µg/ml mitomycin C; 100 mM DTPA) and grown under aeration for 4 h. OD_600_ of the cultures was normalized, bacteria were harvested and luciferase-activity was measured in bacterial lysates. Relative luminescence units (RLU) per unit OD_600_ are indicated. (**B**) Overnight cultures of Ec^MG1655^ as well as Ec^MG1655 Δ*cirA*^ were re-inoculated 1∶20 in fresh LB with indicated supplements (0.25 µg/ml mitomycin C; 100 mM DTPA) and grown under aeration for 4 h. Cultures were normalized to OD_600_, bacteria were harvested and CirA was detected in bacterial lysates by immunoblot using a rabbit-α-CirA antiserum. *E. coli* cytoplasmic protein DnaK was detected as loading control.

**Figure 5:**
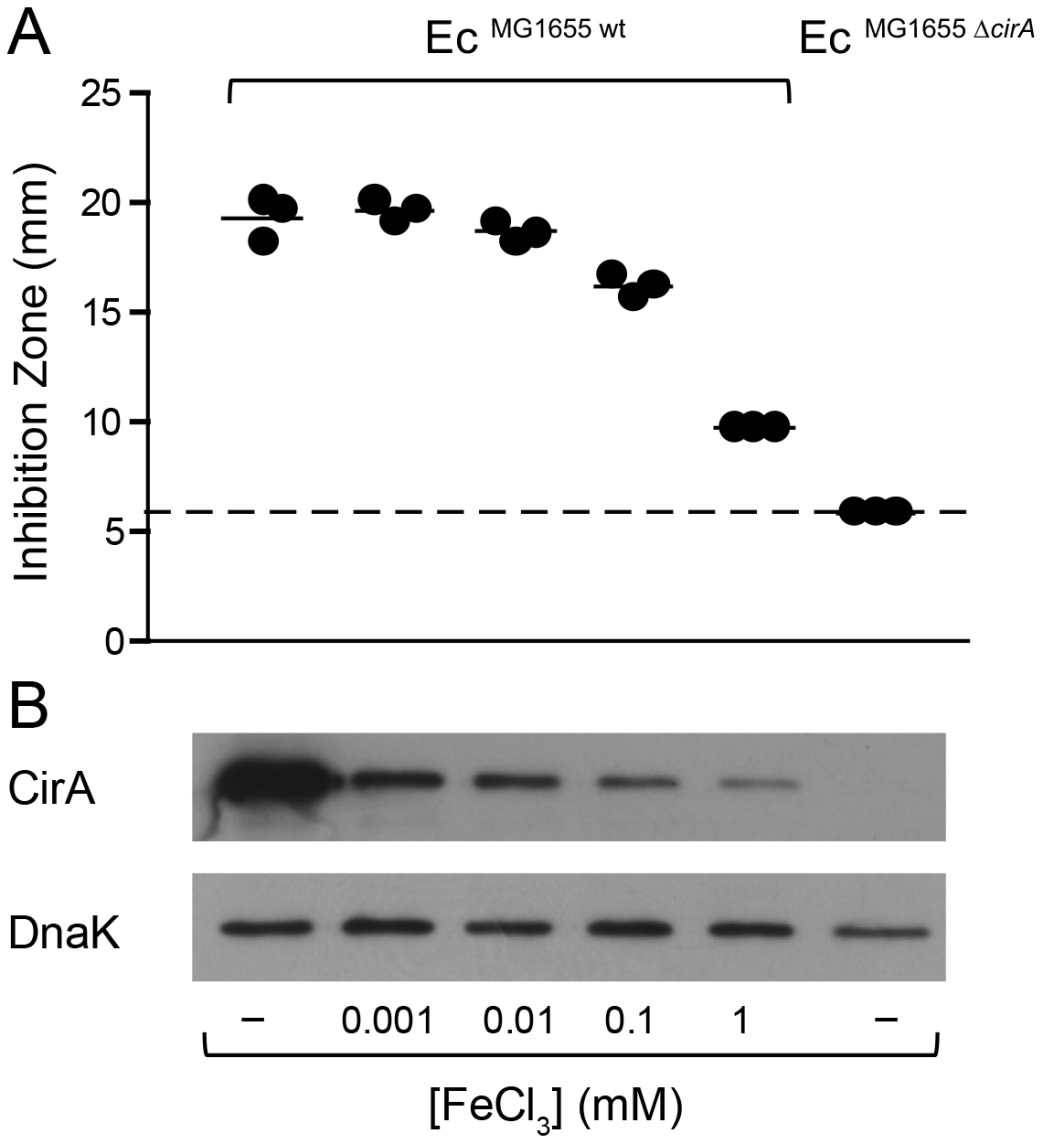
Induction of *cirA* expression increases sensitivity to ColIb of *E. coli*. (**A**) Ec^MG1655^ and Ec^MG1655 Δ*cirA*^ were cultivated in M9 medium o.n., mixed with soft agar and indicated concentrations of FeCl_3_ and overlaid on M9 agar plates. Paper discs with recombinant ColIb were placed on the agar plate and the diameter of the inhibition zone (halo) was measured after 24 h. The detection limit (dotted line) is the size of the paper-disc (6 mm). (**B**) *cirA* expression of *E. coli* grown *in vitro* in M9 medium supplemented with different concentrations of FeCl_3_. Overnight cultures of Ec^MG1655^ and Ec^MG1655 Δ*cirA*^ grown in M9 medium for 12 h were used for inoculation of 2 ml M9 medium supplemented with 1 µM, 10 µM, 0.1 mM or 1 mM FeCl_3_ and subcultured for 7 h. From each subculture, 250 µl (for an OD_600_ of 1) was taken, spun down at 4°C, 10 min, 10, 000 rpm. CirA was detected by immunoblot in bacterial lysates using affinity-purified rabbit-α-CirA-antiserum. *E. coli* DnaK was detected as loading control.

The outer membrane protein CirA is the receptor ColIa and ColIb [18], [19], [20]. Expression of *cirA* is under negative control of Fur [39] (Figure S3). To confirm Fe^III^-dependent regulation of the *cirA* expression in Ec^MG1655^, we generated a *cirA* promoter-*luc*-reporter as well as a polyclonal rabbit-α-CirA antiserum. *CirA* expression was strongly upregulated in LB media upon addition of 100 µM DTPA but not by mitomycin C as confirmed by luciferase assay and immunoblot (Figure 4A, B). This confirmed that Ec^MG1655^ *cirA* was de-repressed in response to Fe^III^-starvation. Next, we tested if *cirA*-expression correlated with sensitivity to ColIb-mediated killing. As expected, Ec^MG1655 Δ*cirA*^ was resistant to ColIb (Figure 5A). This phenotype was complemented by expressing *cirA* on a plasmid in Ec^MG1655 Δ*cirA*^ (Figure S1C). We further investigated, whether successive iron depletion would increase ColIb sensitivity of Ec^MG1655^. To this end, we performed ColIb killing assays of Ec^MG1655^ in M9 minimal media with different concentrations of FeCl_3_ using the same amounts of purified recombinant ColIb. Indeed, Ec^MG1655^ was most sensitive to ColIb in M9 without FeCl_3_ addition and became less susceptible upon FeCl_3_ supplementation (Figure 5A). This correlated with increased *cirA* expression under this condition, as determined by immunoblot (Figure 5B). Thus, sensitivity of Ec^MG1655^ to ColIb increases with elevated *cirA* expression.

### ColIb-dependent competition of *S.* Tm and Ec^MG1655^ *in vitro* is boosted by iron starvation and SOS-stress

**Figure 6:**
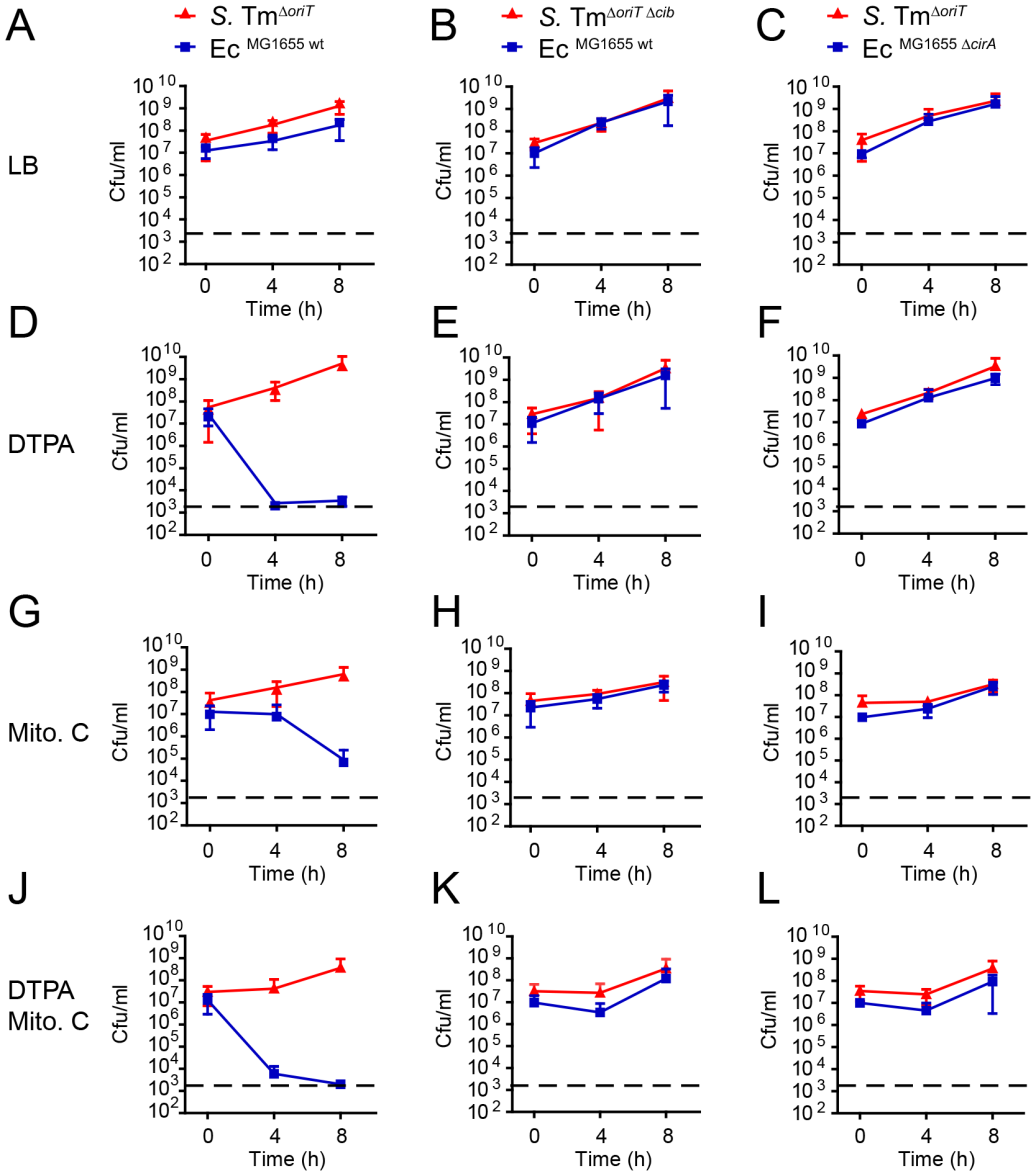
ColIb dependent competition of *S.* Tm and *E. coli in vitro*. Overnight cultures of *S.* Tm^Δ*oriT*^ and Ec^MG1655^ (**A, D, G, J**), *S.* Tm^Δ*oriT* Δ*cib*^ and Ec^MG1655^ (**B, E, H, K**) or *S.* Tm^Δ*oriT*^ and Ec^MG1655 Δ*cirA*^ (**C, F, I, L**) were diluted and normalized to an OD_600_ of 0.05 for each strain in fresh LB with indicated supplements (0.25 µg/ml mitomycin C (Mito. C); 100 mM DTPA). Cfu/ml of both strains were determined at 0 h, 4 h, and 8 h after the start of the subculture. Red lines: *S.* Tm strains, blue lines: *E. coli* strains. Dotted line: detection limit (2000 cfu/ml).

To underscore the importance of environmental conditions for ColIb-dependent competition of *S.* Tm^Δ*oriT*^ and Ec^MG1655^, we performed *in vitro* co-culture experiments. We followed growth of *S.* Tm^Δ*oriT*^ and Ec^MG1655^ and the respective mutants (*S.* Tm^Δ*oriT* Δ*cib*^ and Ec^MG1655 Δ*cirA*^) in co-cultures under different conditions (Figure 6). In the absence of supplements, *S.* Tm^Δ*oriT*^ and Ec^MG1655^ grew at similar rate. *S.* Tm^Δ*oriT*^ outcompeted Ec^MG1655^ by ∼7-fold after 8 h (mean titer *S.* Tm^Δ*oriT*^: 1.7×10^9^ cfu/ml and Ec^MG1655^: 2.3×10^8^ cfu/ml; Figure 6A). In contrast, Ec^MG1655^ was outcompeted by several orders of magnitude after 8 h when either DTPA (7×10^7^-fold), mitomycin C (1×10^4^-fold) or both supplements (6×10^6^-fold) were added to the co-culture (Figure 6D,G,J). *S.* Tm overgrowth *in vitro* was indeed ColIb-dependent, as no killing was observed in the absence of ColIb (*S.* Tm^Δ*oriT* Δ*cib*^) or CirA (Ec^MG1655 Δ*cirA*^) (Figure 5B,C,E–L). Of note, addition of 100 µM DTPA led to more pronounced killing of Ec^MG1655^ than mitomycin C with comparable amounts of ColIb (Figure 3D), suggesting that iron depletion has a greater impact on ColIb-dependent competition (i.e. by enhancing Ec^MG1655^ *cirA* expression and thereby its susceptibility to ColIb). The mutant phenotypes of the *S.* Tm *cib* mutant as well as the Ec^MG1655^ *cirA* mutant were complemented using a plasmid-based complementation approach (Figure S1, Figure S4 and Figure S5).

### Inflammation-induced *Enterobacterial* blooms foster *cib* and *cirA* expression *in vivo*

**Figure 7:**
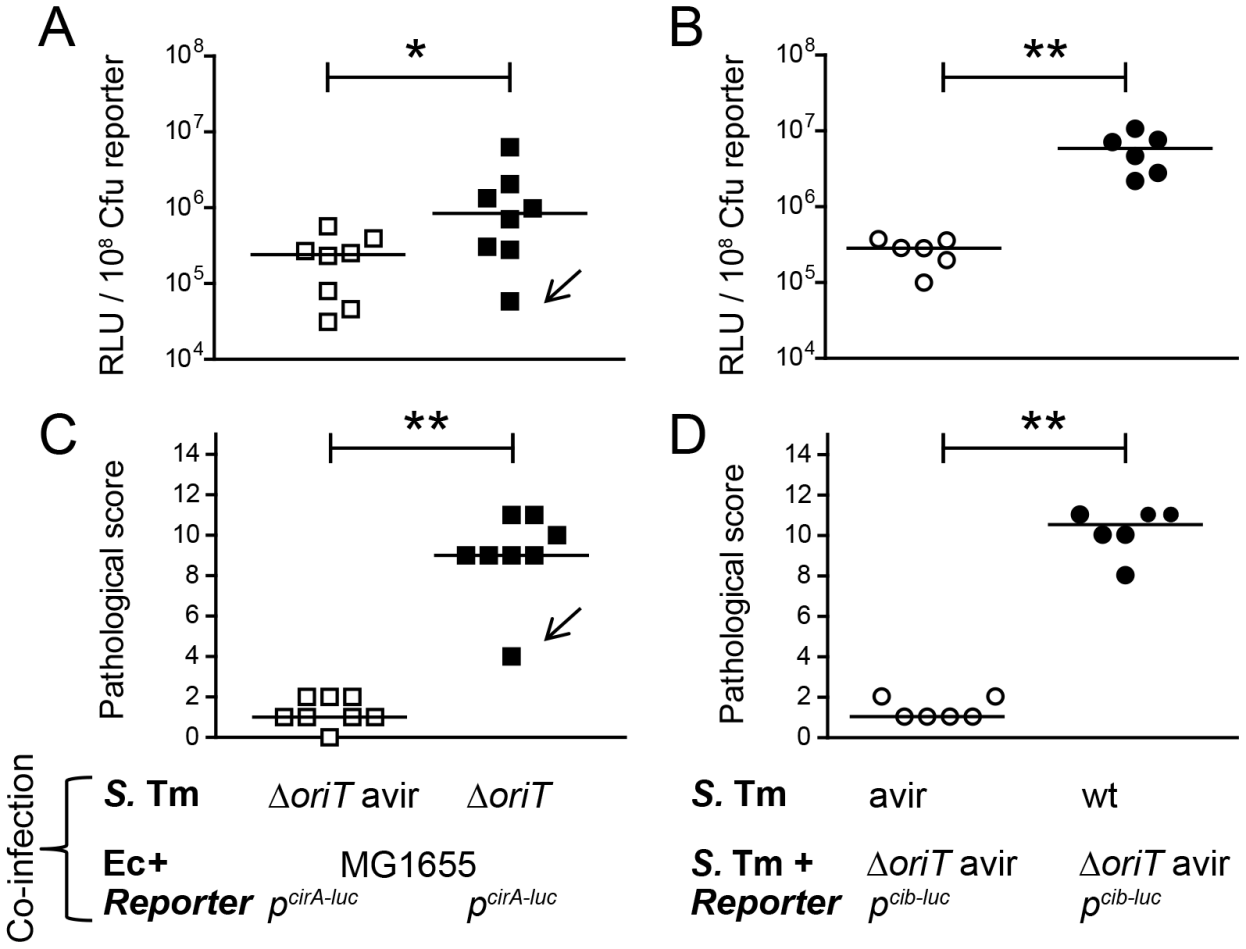
*S.* Tm ColIb and the *E. coli* ColIb receptor CirA are induced in the inflamed gut. To measure *in vivo* regulation of *E. coli cirA* expression, streptomycin-treated LCM-mice were co-infected with 1∶1 mixtures of the luciferase-reporter strain Ec^MG1655^ p*^cirA^* ^-*luc*^ and *S.* Tm^Δ*oriT* avir^ (avirulent) or *S.* Tm^Δ*oriT*^ (wildtype), respectively (**A, C**). To measure *in vivo* regulation of *S.* Tm *cib* expression, streptomycin-treated LCM-mice were co-infected with the luciferase-reporter strain *S.* Tm^Δ*oriT*^ p*^cib^* ^-*luc*^ and *S.* Tm^avir^ (avirulent) or with *S.* Tm^wt^ (wildtype), respectively (**B, D**). Bacteria were harvested from cecal content and luciferase-activity was measured in cecum content (**A, B**). Relative luminescence units (RLU) per 10^8^ cfu of the reporter strain are indicated (**C, D**). Gut inflammation as determined by pathological score of cecal tissue sections of the infected mice. 1–3: no pathological changes; 4–7: moderate inflammation; above 8: severe inflammation. Bars show the median. Arrow in (A) and (C) point at one animal with atypically mild inflammatory symptoms.

**Figure 8:**
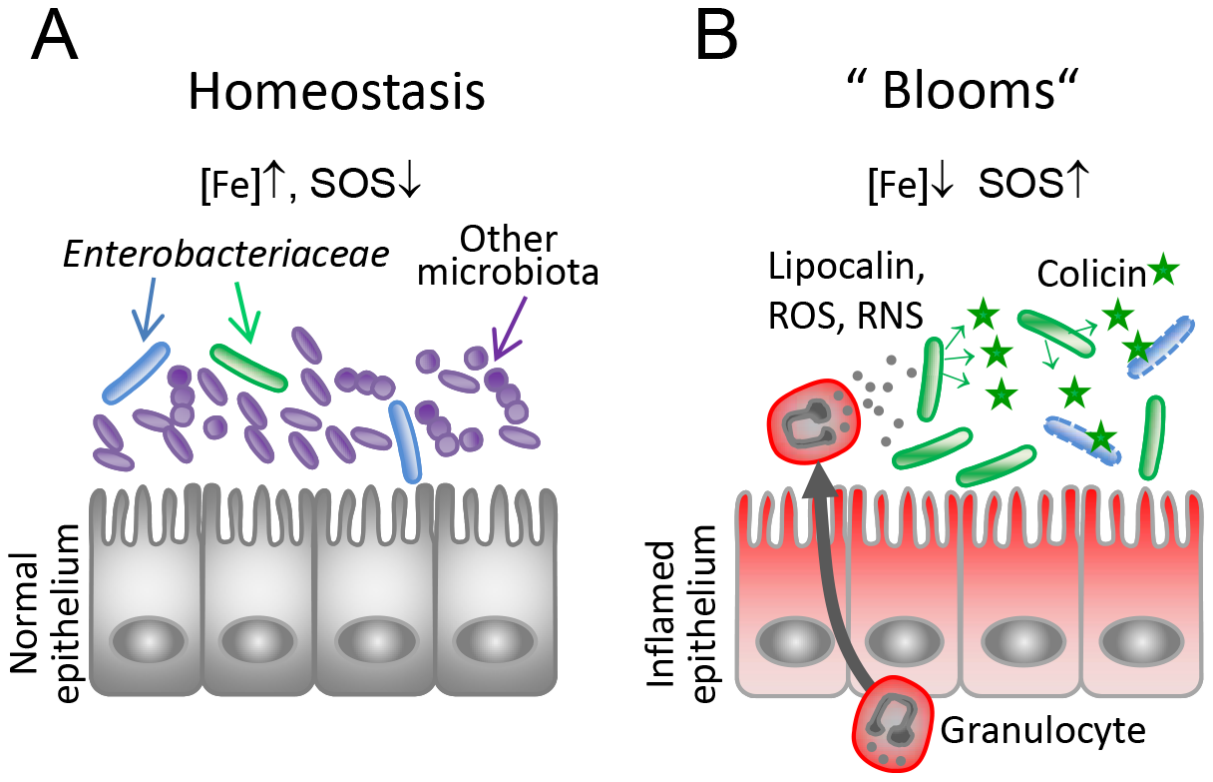
Model for the role of colicins for bacterial competition in inflammation-induced blooms. (**A**) Under homeostatic conditions, *Enterobacteriaceae* (blue, green) are reduced in numbers as they are kept in check by the obligate anaerobic microbiota (violet-blue). Under this condition, colicin expression as well as expression of the colicin surface receptors are relatively repressed (high iron, no triggers of the SOS response). (**B**) Upon induction of an inflammatory response, gut microbial ecology is altered leading to *Enterobacterial* blooms. Neutrophils transmigrate into the gut lumen and produce iron-depleting agents (lipocalin, lactoferrin) and reactive oxygen and nitrogen species (ROS, RNS). This triggers SOS-and Fur-dependent transcriptional responses in *Enterobacteriaceae* and colicin- and colicin-receptor expression is induced. Thereby, the inflammatory response drives colicin-dependent enterobacterial competition.

The *in vitro* co-culture experiments of *S.* Tm and Ec^MG1655^ showed that under iron excess conditions and in the absence of triggers of the SOS response, ColIb confers little to no detectable benefit to *S.* Tm. In contrast, under Fe^III^-limitation or in the presence of stressors, *S.* Tm outcompetes *E. coli*, which is dependent on ColIb production by *S.* Tm and *cirA* expression by *E. coli*. Based on these data, we reasoned that ColIb-dependent competition of *S.* Tm and Ec^MG1655^ in the inflamed gut could indeed be due to increased production of ColIb by *S.* Tm, or up-regulation of the colicin receptor CirA by *E. coli* or both. To address this, we analyzed expression of Ec^MG1655^ *cirA* and *S.* Tm ColIb (*cib*) in the streptomycin mouse colitis model using *firefly-luciferase* reporter-constructs. To generate inflammatory and non-inflammatory conditions in the intestine, LCM mice were infected either with virulent (*S*. Tm^Δ*oriT*^ or *S.* Tm^wt^; +inflammation) or avirulent (*S.* Tm^Δ*oriT* avir^ or *S.* Tm^avir^; -inflammation) *Salmonella* strains, respectively. To investigate regulation of Ec^MG1655^ *cirA* expression, mice were co-infected with Ec^MG1655^ carrying the p*^cirA^* ^-*luc*^-reporter plasmid (Figure 7A,C). To investigate regulation of *S.* Tm *cib* expression, mice were co-infected with *S.* Tm^Δ*oriT avir*^ carrying the p*^cib^* ^-*luc*^-reporter plasmid (Figure 7B,D). The experiments showed that luciferase levels for both the p*^cirA^* ^-*luc*^- and the p*^cib^* ^-*luc*^-reporters were significantly increased in the inflamed intestine (Figure 7A–D). Therefore, these data are in agreement with our initial hypothesis and suggest that in response to gut inflammation, ColIb production by *S.* Tm and susceptibility of Ec^MG1655^ to ColIb are increased. This explains how inflammation fuels ColIb dependent competition of *S.* Tm and commensal *E. coli* (Figure 8).

## Discussion

### *Enterobacterial* blooms provide environmental cues for colicin production and susceptibility

Inflammatory conditions in the gut shape gut microbial community structure and are characterized by an increased prevalence of facultative anaerobic bacteria (“blooms”), in particular members of the *Enterobacteriaceae* family [4], [8], [40]. Commensal *E. coli* strains can hitchhike gut inflammation induced by inflammatory bowel diseases or enteric pathogens (*S.* Tm or *Citrobacter rodentium*) [5], [7], [12]. Commensal and pathogenic representatives of the *Enterobacteriaceae* efficiently dwell in blooms as they can exploit resources with increased abundance in the inflamed gut (ethanolamine, the anaerobic electron-acceptors tetrathionate and nitrate) [11], [12], [41]. Up to 15 different *E. coli* strains can be detected in one individual human gut ecosystem [42]. Thus, successful competition for resources should be of major importance for bacteria in order to come out on top and eventually benefit from an episode of gut inflammation [8]. Here, we propose that colicins such as ColIb are effective means to fight off competing bacteria, particularly in inflammation-induced enterobacterial blooms.

Which environmental cues promote ColIb-dependent bacterial competition in blooms? As an immediate inflammatory defence reaction, the host deprives potential invading pathogens of nutritionally-required iron. Neutrophils release iron-sequestering lactoferrin upon degranulation [43]. Further, lipocalin 2 (LCN2), an antimicrobial protein binding the bacterial siderophore enterochelin [44] is abundantly expressed by neutrophils and intestinal epithelial cells. LCN2 is produced in response to infection with wildtype but not avirulent *S.* Tm strains in the streptomycin-colitis model [9]. *S.* Tm overcomes LCN2-imposed inhibition by production of salmochelin [45], [46], a siderophore resistant to binding by LCN2. Salmochelin is produced in a Fur-dependent manner in response to iron limitation and upregulated by *S.* Tm thriving in the inflamed gut [10]. Therefore, host-mediated iron restriction in the inflamed gut poses an environmental cue for inducing ColIb production by *S.* Tm. Other cues for ColIb production are compounds triggering the SOS response. Yet, the exact source and identity of those compounds in the inflamed gut is ill-defined. The SOS response in *Enterobacteriaceae* can be triggered by antibiotics which directly affect DNA integrity (mitomycin C), DNA replication via the DNA gyrase (quinolones) or induce membrane stress (β-lactams) [28], [47]. Further, oxidative stress induced by reactive oxygen species (ROS), superoxides, H_2_O_2_ or free radicals formed by UV-light induce DNA damage and thereby the SOS response [28], [48]. Neutrophils infiltrating the intestinal lumen in response to *Salmonella* infection as well as activated iNOS-producing epithelial cells likely represent a source of free radicals and potential inducer of the SOS response [40], [49]. Recently, it was demonstrated that *E. coli* indeed upregulates stress-induced proteins such as GroL, RecA, YggE and the Fe-S cluster repair protein NfuA in the inflamed gut [50].

Not only was the expression of *cib* increased in the inflamed gut, but also the corresponding surface receptor *cirA*. CirA is the receptor for monomers, dimers and linear trimers of 2,3-dihydroxybenzoylserine, breakdown products of enterochelin [51]. Further, it was shown, that *cirA* mutants are attenuated in the uptake of monomeric catechol and its analogues [52]. In order to avoid iron overload and its negative consequences (e.g. the formation of hydroxyl-radicals), iron-uptake systems are tightly controlled at the transcriptional level and only de-repressed under iron-limiting conditions. *In vitro*, susceptibility to ColIb of *E. coli* was drastically increased under Fe^III^-depleted conditions, which correlated with elevated CirA protein levels. In the same way, the *in vitro* competition experiments suggest that *S.* Tm benefits most from ColIb production under iron-limiting conditions (7×10^7^-fold). The growth advantage was lower in the presence of mitomycin C (1×10^4^-fold) or under both iron limitation and mitomycin C (6×10^6^-fold). Of note, the amount of free *S.* Tm ColIb in the medium was even higher in the presence of mitomycin C, than with addition of DTPA only (Figure 3D), supporting the idea, that the expression level of *cirA* has a higher impact on the competition, compared to that of ColIb (Figure 6D,G). Hitherto, the underlying mechanism of ColIb-release or secretion triggered by the SOS response is not known. In conclusion, we reason that increased *cirA* expression and consequential high susceptibility of *E. coli* to ColIb may explain for the most part colicin-dependent competition in blooms (Figure 8).

Interestingly, several other colicins parasitize siderophore outer membrane receptors, which are all under control of the Fur-regulon. ColM binds to the ferrichrome receptor FhuA while ColB and ColD bind to FepA, the receptor for enterobactin [15]. This suggests that increased sensitivity to colicin-mediated killing under iron depletion may also apply for other colicins binding to TonB-dependent outer-membrane transporters. Previously, it was shown that killing of susceptible bacteria by pyocin, a bacteriocin produced by *Pseudomomas* spp., is increased under iron-limited conditions [53]. Pyocin binds the ferri-pyoverdine receptor FpvA, which is controlled by Fur. In summary, physiological changes of the murine intestine upon *Salmonella*-induced colitis are likely to provide the environmental cues required for upregulation of both, ColIb and its receptor CirA. Yet, we cannot rule out that other physiological parameters altered in the inflamed gut also contribute to the observed phenotype. So far, it is unclear if expression of other types of colicins as well as other *Enterobacteriaceae*-derived bacteriocins (microcins, pyocins, klebicins) would be upregulated in the inflamed gut. The majority of these bacteriocins is only under control of the SOS response and repressed by LexA and not regulated in a Fur-dependent fashion. Thus, it remains to be shown if the principle of colicin-colicin-receptor upregulation in the inflamed gut also applies to other bacteriocins and their respective receptors.

### Colicin-dependent bacterial competition is enhanced in the inflamed gut

Supposedly, colicins play a major role in mediating bacterial population dynamics [54]. Superior fitness of colicinogenic over sensitive strains *in vivo* could so far only be demonstrated in few studies performing long-term competitive infection experiments (≥12 weeks) [30], [31]. In contrast, a number of other studies reported that high bacteriocidal activity against closely related, sensitive strains observed *in vitro* could not be recapitulated in *in vivo* experiments (see below). Our data presented in this paper might explain this puzzling observation: we suggest that the fitness benefit of colicin production strongly depends on the environmental conditions prevailing in the gut. Under normal conditions, colicin expression and expression of their cognate receptors may not be stimulated enough to induce colicin-dependent inhibition of the sensitive strain. In the absence of gut inflammation, *S.* Tm did not benefit from ColIb in competition with *E. coli*. Likewise, competition experiments in germfree mice with a colicin-producing *E. coli* and a sensitive strain resulted in equal colonization levels of both strains over weeks (no inflammation induced) [32], [33], [34] and similar results were obtained for other strains and colicins [55], [56]. The underlying reasons for the absence of an overt fitness-benefit of colicin production were attributed to colicin inactivation by intestinal proteases [33], acquisition of colicin-resistances [55] or absent colicin activity under anaerobic conditions [35], [36]. Our study suggests that absence of inflammatory conditions might be an additional explanation.

### Implications for the evolution of colicin-dependent competition

Colicin production is a common trait in *E. coli* populations [54]. On average, 30% of natural *E. coli* populations produce one or more colicins [57]. Many experimental and theoretical studies have addressed the ecological consequences of colicin production in bacterial populations [58], [59]. In general, it is assumed, that colicins afford a competitive advantage to the strain producing it. However, the producer pays a fitness cost due to the higher metabolic load of colicin synthesis as well as lethality of production (e.g. lysis-mediated colicin release). Bacteria have partially overcome this limitation by applying the principle of ‘division of labor’. In a population of colicin producers, only a small fraction of bacteria are induced to produce the colicin ( = phenotypic heterogeneity) [60], [61]. In *recA* negative strains decreased frequencies of colicin producers were observed, suggesting that the rate of colicin production is regulated by the SOS response [60]. This strategy seems to be evolutionary successful, as colicins released by the subpopulation serve as a “common good” for the whole population and secure propagation of the shared genotype. Nevertheless, colicin expression needs to be tightly controlled to ensure, that the fraction of producers is kept at low rates under conditions, when colicin is not required. Those conditions include the lack of stress, nutrient starvation but also the absence of any direct competitors.

Thus, we assume that colicin production of a bacterial population should be confined to environmental niches which are characterized by high density and diversity of competing *E. coli* and close relatives. *E. coli* titers in the mammalian gut lumen are rather low as the intestinal tract is dominated by strictly anaerobic bacteria [62]. *E. coli* predominantly colonizes the mucus layer of the large intestine where it thrives on mucin-derived sugars [63]. Thus, the intestinal mucus layer is one highly competitive environment for *E. coli* and colicins may be beneficial for competing for the preferred limiting substrates [64]. In contrast, we identify inflammation-induced blooms as an alternative niche for colicin-dependent *Enterobacterial* competition (Figure 8). *Enterobacterial* blooms can contain multiple closely-related species at high concentrations which likely compete for the same resources. Under this highly competitive situation, the chances are increased that colicin-sensitive competitors are present at high numbers. Thus, the bacteria may benefit hugely from colicin production under this environmental condition. Moreover, the population size of the colicin-producer is large enough to tolerate loss of a fraction of the population due to suicidal colicin production. In summary, the results presented here provide evidence that intestinal inflammation drives colicin-dependent competition by bacteria of the *Enterobacteriaceae* family. These findings shed new light on the role of colicins as important fitness factors providing a competitive advantage for growth in *Enterobacterial* blooms.

## Materials and Methods

### Ethics statement

All animal experiments were approved by the Regierung von Oberbayern and the Kantonales Veterinäramt Zürich performed according to national German and Swiss guidelines (Deutsches TschG; Schweizer Kantonale TschV). The permit no. 55.2-1-54-2532-49-11 (Germany) and 201/2007 (Switzerland).

### Animal experiments

All mice used in the study were on C57BL/6J background and bred at the Rodent Center, ETH Zürich and the Max-von-Pettenkofer Institute, LMU Munich under SPF conditions in individually ventilated cages. Low-complexity microbiota (LCM) mice were generated by associating germfree mice with members of the Altered Schaedler flora [65] as described previously [66]. Conventional SPF C57BL/6J mice were purchased from Janvier, Le Genest Saint Isle. For infections, conventional and LCM mice were pretreated with streptomycin and infected by gavage with 5×10^7^ cfu *S.* Tm or mixtures of *S.* Tm and *E. coli* as described [7]. For *in vivo* luciferase-assays, LCM mice were pretreated with ampicillin (20 mg/animal 24 h prior to infection). Live bacterial loads in the cecal content were determined by plating on MacConkey-agar (Roth) with respective supplements (streptomycin 100 µg/ml; kanamycin 30 µg/ml; chloramphenicol 30 µg/ml; ampicillin 100 µg/ml and tetracycline 12,5 µg/ml). Histology of the cecum was done at necropsy. Cecum tissue was embedded in O.C.T. (Sakura, Torrance) and flash frozen. Cryosections (5 µm) of the cecal tissue were H&E-stained and scored as described in detail in [6]. The parameters submucosal edema, PMN infiltration, loss of goblet cells and epithelial damage were scored according to the severity of inflammatory symptoms yielding a total score of 0–13 points. For infections, *E. coli* and *S.* Tm strains were grown as described [67]. Briefly, cultures in LB supplemented with 0.3M NaCl were inoculated with 2–3 bacterial colonies from plates. Bacteria were grown over night for 12 h and subcultures (1∶20) for an additional 4 h. Bacteria were mixed (as indicated) washed in PBS and applied to the mice in a total volume of 50 µl by gavage.

### Construction of bacterial mutants and plasmids

Bacterial strains and plasmids used in this study are listed in **Table 1**. Ec^MG1655 Δ*cirA*^ (LPN2) was constructed using the lambda Red recombinase system as described using pKD4 as template for the kanamycin-resistance gene including the FRT-sites [68]. Briefly, Ec^MG1655^ was transformed with the plasmid pKD46. The kanamycin resistance cassette from plasmid pKD4 was amplified by PCR using primers K12Δ*cirA*_Fwd/K12Δ*cirA*_Re (**Table S1**). Correct recombination was verified by PCR using primers *cirA*-up/*cirA*-down and *cirA*-up/*cirA*-d1 (**Table S1**). *S.* Tm^avir Δ*oriT*^ (LPN5) was generated by P22-transduction of the Δ*oriTnikA*::cat allele from M1407 into M557 [37]. Correct insertion was verified by PCR using primers *ΔoriTnikA*rev_val, Δ*oriTnikA_*val. The P2 plasmid was cured from *S.* Tm Δ*invG*; *sseD::aphT* cured as described previously [7]. For the generation of c-terminal CirA-His-tag fusion, the open reading frame of *cirA* was amplified from *E coli* Nissle genomic DNA by PCR, using Fow_*cirA_Nhe*I *and* Re_*cirA_Xho*I primers (**Table S1**) and cloned into pET-24c (Novagen) via *Nhe*I *and Xho*I to yield pLPN13. For the generation of c-terminal ColIb-His-tag fusion, the ColIb gene *cib* was amplified from *S.* Tm^wt^ genomic DNA by PCR, using primers Fow*_colicin_Nhe*I and Re_*colicin_Xho*I (**Table S1**) and cloned into pET-24c via *Nhe*I and *Xho*I to yield pLPN14. To generate pLPN1, the *cirA* promoter was amplified from *E. coli* ^Nissle^ using *pcirA-BamH*I, *pcirA-Xba*I (**Table S1**) and inserted in *Bam*HI and *Xba*I digested pM979 [69]. For generation of pM1437, the *cib* promoter from *E. coli* ^8178^ was amplified using pColIb-*Xba*I, pColIb-*Bam*HI (**Table S1**) and inserted in pM968 [69] via restriction with *Xba*I and *Bam*HI. To generate pLPN15 and pLPN16, the firefly-luciferase gene *luc* from pLB02 [70] was amplified with Luc-for-*Bam*HI and Luc-rev-*Hind*III primers (**Table S1**) and inserted into *Bam*HI/*Hind*III digested pLPN1 or pM1437, respectively.

Primers pWSK29-Gbs-for and pWSK29-Gbs-rev were used in a PCR with pWSK29 [71] as a template to amplify the low-copy-number plasmid (**Table S1**). Primers CirA-pWSK29-Gbs-for and CirA-pWSK29-Gbs-rev were used in a PCR with chromosomal DNA of Ec^MG1655^ as a template to amplify *cirA* including its natural promoter. Primers Cib-Imm-pWSK29-Gbs-for and Cib-Imm-pWSK29-Gbs-rev were used in a PCR with *S.* Typhimurium strain SL1344 plasmid pCol1B9_SL1344 as a template to amplify the *cib*/*imm* locus including both natural promoters. The pWSK29 PCR fragment was combined with the *cirA* or *cib*/*imm* fragment in a Gibson assembly reaction [72]. Four microliters of the Gibson assembly mix were transformed into chemically competent *E. coli* Mach1 T1 cells (Life Technologies). Constructs were verified using colony PCR, restriction analysis and sequencing.

### Identification of regulator binding sites

For annotation of transcription factor binding sites (Fur and LexA regulon), all known transcription factor binding sites of each family one were taken from RegulonDB (version 8.0) [73] and a binding motif was created using MEME [74]. The nucleotide sequences of the *cib* (*S.* Tm SL1344; EMBL accession no. FQ312003) and *cirA* promoter regions (*E. coli* MG1655 genome accession no. NC_000913.2) were searched for the computed MEME binding site motifs using MAST [74].

### Generation and affinity purification of recombinant proteins

For expression of ColIb-His, we used *E. coli* BL21 transformed with pC831-2 (expression of the ColIb immunity protein gene *imm* [7]) and pLPN14. For expression of *cirA*-His we used *E. coli* BL21 transformed with pLPN13 (**Table S1**). Over-night cultures of bacteria, grown at 37°C, 180 rpm in Luria-Bertani (LB) medium containing antibiotics were used for inoculation of subcultures (dilution 1∶20). At OD_600_ between 0.6–0.8 the subcultures was induced with 0.1 mM isopropyl β –D-thiogalactopyranoside (IPTG) and incubated for additional 4 h at 37°C, 180 rpm. Bacteria were harvested (4,500 rpm, 30 min at 4°C), resuspended in 40 ml 1×PBS and spun down at 5,000 rpm, 20 min at 4°C. The pellet was frozen at −20°C. Thereafter, the pellet was thawed and resuspended in 25 ml loading buffer (40 mM Na_2_HPO_4_, 0.3M NaCl, 5 mM Imidazol, pH 7.8), supplemented with 2 mM phenylmethylsulfonyl fluoride (PMSF) and benzonase nuclease (Novagen). Bacteria were lysed in the French Press (1,000 PSI) and the lysate was filtered (0.22 µm). Further, the lysate was loaded on a 5 ml HisTrap column (GE Healthcare), and purified using the ÄKTA system (GE Healthcare). ColIb-His was eluted with 5 mM Imidazole. The fractions containing the protein were desalted on a 5 ml HiTrap desalting column (GE Healthcare), using the ÄKTA system and exchange buffer (20 mM Na_2_HPO_4,_ 100 mM NaCl, pH 7.4). CirA-His was purified as outlined above for ColIb-His, but with following exceptions: loading buffer for the French Press (8M Urea, 40 mM Na_2_HPO_4_, 0.3M NaCl, 5 mM Imidazol, pH 7.8, 2 mM PMSF, and Benzonase nuclease): loading buffer for HisTrap column (6M Urea, 40 mM Na_2_HPO_4_, 0.3M NaCl, 5 mM Imidazol, pH 7.8); exchange buffer (4M Urea, 20 mM Na_2_HPO_4,_ 100 mM NaCl, pH 7.4). Rabbit antisera against ColIb-His and CirA-His were raised using standard protocols (Pineda Antikörper-Service, Berlin, Germany). 6 mg/ml ColIb-His (in 20 mM Na_2_HPO_4_, 100 mM NaCl, pH 7.4) and 6 mg/ml CirA-His (in 20 mM Na_2_HPO_4_, 100 mM NaCl, 4M Urea, pH 7.4) were used for immunization. Control and immune serum were received from bleedings day 61, 90 and 135 post immunization.

### Affinity purification of rabbit-antisera

Affinity purification of polyclonal rabbit α-ColIb-His antiserum was done using the Aminolink kit (Thermo Scientific) following the manufacturer's protocol with some minor modifications. For ColIb-His, PBS was used as binding/wash buffer and 1M Glycine, pH 2.7 was used as elution buffer. ColIb-His (stored in 20 mM Na_2_HPO_4_, 100 mM NaCl, pH 7.4) was added to the binding buffer at 1∶3 ratio. Desalting of the affinity-purified rabbit-α-ColIb-His antiserum was done using PD-10 desalting columns with PBS as exchange buffer (GE Healthcare). For CirA-His, His-tagged CirA (20 mM Na_2_HPO_4_, 100 mM NaCl, 4M Urea, pH 7.4) was dialyzed against PBS using 5 ml Zebra Spin desalting columns (Thermo scientific) shortly before coupling to the column. Coupling was done with CirA-His (in PBS) and coupling buffer supplemented with 4M Urea (MP Biomedicals). Desalting of the affinity-purified rabbit-α-CirA-His was done with Zebra Spin desalting columns with PBS containing 0.05% sodium azide (Merk). Purified antisera were stored at −80°C with addition of sodium azide to 0.01%.

### Colicin killing-assay

For measuring colicin production and -sensitivity, the colicin-producing strain was grown o.n. as small spot (ø5 mm) on LB agar containing 0.25 µg/ml mitomycin C (Roth). The plate was overlaid with the tester strain in top-agar (0.75% agar). Growth of the tester strain was analyzed after 24 h. Formation of an inhibition zone (halo) around the producer indicated production of colicin and sensitivity of the tester strain. For determining ColIb sensitivity dependent on the Fe^III^ concentration, the assay was modified accordingly. Starter cultures of *E. coli* in 3 ml M9 medium (40 mM Na_2_HPO_4_×2H_2_O, 20 mM KH_2_PO_4,_ 9 mM NaCl, 2 g/L NH_4_Cl, 1 mM MgSO_4_, 100 µM CaCl_2,_ 2 g/l D-glucose, 10 mg/l thiamine, 500 mg/l histidine) were grown for 10 h and used for inoculation (1∶20) of 2 ml M9 medium, supplemented with 1 µM, 10 µM, 0.1 mM or 1 mM FeCl_3_ and grown for 12 h. From each subculture, 50 µl was added to 5 ml 50°C 0.7% M9 top-agar (0.75% agar), which was used to overlay M9 agar plates. Further, antimicrobial susceptibility test discs (Oxoid) were laid on each plate and supplemented with 8 µl 1.3 mg/ml recombinant His-tagged ColIb. The plates were incubated over-night at 37°C.

### Growth of bacterial strains for *in vitro* assays

Bacteria were grown in a starter culture in LB or M9 media and used for inoculation of subcultures (1∶20), except of *in vitro* co-cultures, where subcultures were inculcated to an OD_600_ of 0.05 for each strain. Following supplements were used: 0.25 µg/ml mitomycin C (Roth); 100 µM diethylenetriaminepentaacetic acid (DTPA; Sigma), 1 µM, 10 µM, 0.1 mM or 1 mM FeCl_3_ (Sigma). All cultures were grown at 37°C on a wheel rotor, except of *in vitro* co-cultures, where subcultures were grown in Erlenmeyer-flasks in a shaker at 200 rpm.

### Luciferase assay

Luciferase assays were performed as described [75]. Briefly, overnight cultures (3 ml LB, 100 µg/ml ampicillin) were grown for 12 h and used for 1∶20 inoculation of 3 ml subcultures (LB, 100 µg/ml ampicillin with respective supplements) and grown for 4 h. From each subculture, 250 µl (of an OD_600_ of 1) was spun down for 5 min, 14,000 rpm, 4°C. The supernatant was removed and the bacterial pellet was frozen at −80°C for 1 h. Further, the pellet was thawn and resuspended in 500 µl lysis buffer (100 mM K_2_HPO_4_/KH_2_PO_4_ buffer, pH 7.8, 2 mM EDTA, 1% Triton X-100, 5 mg/ml BSA, 1 mM DTT and 5 mg/ml lysozyme) and incubated for 15 min at room temperature while vortexing every 3 minutes. Bacterial lysates (25 µl) were transferred in 96-well plates (white; Thermo scientific) and 50 µl luciferase reagent [1 mM (MgCO_3_)_4_Mg(OH)_2_×5H_2_O, 20 mM tricine, 0.1M EDTA, 470 µM D(−) luciferin (Sigma), 530 µM Mg-ATP (Sigma), 125 µM glycylglycine (Sigma), 270 µM Li_3_-coenzym A (Sigma), 33 mM DTT] was added to each well. Luminescence was measured using a FLUOstar Optima plate reader (BMG Labtech).

For luciferase assay from bacteria extracted from cecum content, the cecum content was harvested from infected mice and shortly stored on ice. The cecum content was resuspended in 500 µl PBS (0.1% tergitol) and mixed in a tissue-lyser (Qiagen; 5 min; 50 Hz). Further, the cecum content was filtered through a 40 µm cell-sieve (Milian). Samples were taken to determine the cfu/ml of the reporter strain by plating on MacConkey agar with respective antibiotics. A defined volume (i.e. 900 µl) was pelleted at 4°C, 2 min, 14,000 rpm. The supernatant was removed and the pellet was frozen in dry ice and stored at −80°C. The samples were then thawn and processed as described above. Only values above detection limit (control cecum content) were considered. The relative luminescence units (rlu) per cfu luciferase-reporter strain were calculated.

### Generation of samples for immunoblot

Overnight cultures of 3 ml M9 medium, grown for 12 h were used for inoculation (1∶20) of 2 ml M9 medium supplemented with 1 µM, 10 µM, 0.1 mM or 1 mM FeCl_3_ subculture, grown for 7 h. Starter culture of 3 ml LB, grown for 12 h was used for inoculation of 3 ml LB with supplements grown for 4 h. From each subculture, 250 µl (for an OD_600_ of 1) was taken, spun down at 4°C, 10 min, 10, 000 rpm. The supernatant was removed and bacterial pellet was frozen in liquid nitrogen and thawn at room temperature for 15 min (repeated three times), resuspended in 100 µl lysis buffer (50 mM Tris, pH 7.5, 150 mM NaCl, 5 mM EDTA, 0.25% nonidet P-40, 1 mg/ml lysozyme) and incubated in thermomixer at 550 rpm at 23°C, for 1 h and thereafter spun down at 4°C, 20 min, 14 000 rpm. Total protein was quantified in the lysate using protein assay reagent (BioRad). Further, bacterial lysate was added to protein loading buffer (50 mM Tris, 100 mM DTT, 2% SDS, 0.1% bromphenolblue, 10% glycerol) and incubated for 10 min at 95°C. For supernatant fractions 500 µl (for an OD_600_ of 1) of the subculture were spun down twice, supernatant was added to 5× protein loading buffer and incubated for 10 min at 95°C.

### SDS-polyacrylamide gel electrophoresis (PAGE) and immunoblot

Proteins were separated by SDS gel electrophoresis [76]. Proteins were transferred onto a nitrocellulose membrane (GE Healthcare) at 300 mA for 2 h. The membrane was blocked in PBS (0.1% tween; 5% milk powder) and probed with antisera (affinity-purified α-CirA-His (1∶50) or affinity-purified α-ColIb-His (1∶500). Goat-α-rabbit-HRP (GE-Healthcare) was used as secondary antibody. For detection of *E. coli* and *S.* Tm DnaK, the mouse monoclonal α-*E. coli* DnaK antibody (mAb 8E2/2; Enzo Life Sciences) and a secondary goat-α-mouse-HRP (Sigma) was used. Blots were developed with ECL detection system (GE Healthcare).

### Statistical analysis

Statistical analysis was performed using the exact Mann-Whitney U Test (Graphpad Prism Version 5.01). P-values less than 0.05 (2-tailed) were considered statistically significant.

## Supporting Information

Figure S1 **Halo-assay to confirm phenotypes of ColIb production and susceptibility.** (**A**) ColIb susceptibility of Ec^Nissle^ and Ec^MG1655^. *S.* Tm^wt^ was spotted on LB agar plates containing mitomycin C and incubated o.n. to induce ColIb secretion. Ec^Nissle^ and Ec^MG1655^ were cultivated in LB medium o.n., mixed with soft agar and overlaid on the agar plates. (**B**) Plasmid-based complementation of the ColIb-deficient *S.* Tm mutant *S.* Tm^Δ*oriT* Δ*cib*^. *S.* Tm^Δ*oriT*^, *S.* Tm^Δ*oriT* Δ*cib*^ and *S.* Tm^Δ*oriT* Δ*cib*^ p^compl. *cib*^ were spotted on LB agar plates containing mitomycin C and incubated o.n. to induce ColIb secretion. Ec^MG1655^ was cultivated in LB medium o.n., mixed with soft agar and overlaid on the agar plates. (**C**) Plasmid-based complementation of the CirA-deficient Ec^MG1655 Δ*cirA*^ mutant. *S.* Tm^Δ*oriT*^, and *S.* Tm^Δ*oriT* Δ*cib*^ were spotted on LB agar plates containing mitomycin C and incubated o.n. to induce ColIb secretion. Ec^MG1655 Δ*cirA*^ and Ec^MG1655 Δ*cirA*^ p^compl. *cirA*^ were cultivated in LB medium o.n., mixed with soft agar and overlaid on the agar plates. The experiments were done in triplicates and the diameter of the ColIb inhibition zone (halo) was measured after 24 hours. The detection limit (dotted line) is the average size of the *S.* Tm^wt^ colony.
(PDF)

Figure S2 **Nucleotide sequence of** ***S.*** **Tm^wt^** ***cib imm*** **and its respective promoter regions.** Fur- and LexA repressor binding sites were annotated to the *cib* and *imm* sequence region of *S.* Tm^wt^ as described in the materials and methods section. The position of the Fur-box, LexA-box, major transcription start sites and their corresponding −10 and −35 regions are indicated, as well as the open reading frame and the prospective ribosome-binding site (S.D.).
(PDF)

Figure S3 **Nucleotide sequence of Ec^MG1655^** ***cirA*** **and its promoter region.** Fur-repressor binding site was annotated to the *cirA* sequence region of Ec^MG1655^ as described in the materials and methods section. The position of the Fur-box, major transcription start sites and their corresponding −10 and −35 regions are indicated, as well as the open reading frame and the prospective ribosome-binding site (S.D.).
(PDF)

Figure S4 >**Characterization of plasmid-based complementation of** ***S.*** **Tm^Δ^** ^***oriT* Δ*cib***^ **and Ec^MG1655 Δ^** ^***cirA***^ **mutant strains by western blot.** Overnight cultures of indicated *S.* Tm (**A**) and Ec^MG1655^ strains (**B**) were re-inoculated 1∶20 in fresh LB with indicated supplements (0.25 µg/ml mitomycin C; 100 mM DTPA) and grown under aeration for 4 h. Cultures were normalized to OD_600_, bacteria were harvested and ColIb was detected in bacterial lysates as well as in the culture supernatant by immunoblot using an affinity-purified rabbit-α-ColIb antiserum (**A**). *S.* Tm DnaK was detected as loading control (**A**). Ec^MG1655^ CirA was detected in bacterial lysates by immunoblot using a rabbit-α-CirA antiserum (**B**). *E. coli* cytoplasmic protein DnaK was detected as loading control (**B**).
(PDF)

Figure S5 **ColIb dependent competition of complemented** ***S.*** **Tm and** ***E. coli*** **mutant strains** ***in vitro*** **.** Overnight cultures of *S.* Tm*^ΔoriT^* and Ec^MG1655 Δ*cirA*^ p^compl. *cirA*^ (**A, D, G, J**), *S.* Tm^Δ*oriT* Δ*cib*^ and Ec^MG1655 Δ*cirA*^ p^compl. *cirA*^ (**B, E, H, K**) or *S.* Tm^Δ*oriT* Δ*cib*^ p^compl. *cib*^ and Ec^MG1655^ (**C, F, I, L**) were diluted and normalized to an OD_600_ of 0.05 for each strain in fresh LB with indicated supplements (0.25 µg/ml mitomycin C (Mito. C); 100 mM DTPA). Cfu/ml of both strains were determined at 0 h, 4 h, and 8 h after start of the subculture. Red lines: *S.* Tm strains, blue lines: *E. coli* strains. Dotted line: detection limit (2000 cfu/ml). Plasmid-based reconstitution of *cib* and *cirA* to the mutant strains leads to an over-complementation apparent by ColIb-dependent killing of *E. coli* in LB in the absence of supplements which is attributed to the multi-copy nature of the complementation-plasmid (**A, C**).
(PDF)

Table S1 **Primers used in this study.** All PCR primer sequences used in the study are listed.
(DOCX)
