# Supplementary figures and images for: Inflammation Fuels Colicin Ib-Dependent Competition of Salmonella Serovar Typhimurium and E. coli in Enterobacterial Blooms
Source: PLoS Pathog. 2014 Jan 2;10(1):e1003844. doi: 10.1371/journal.ppat.1003844 (PMC3879352; doi:10.1371/journal.ppat.1003844)

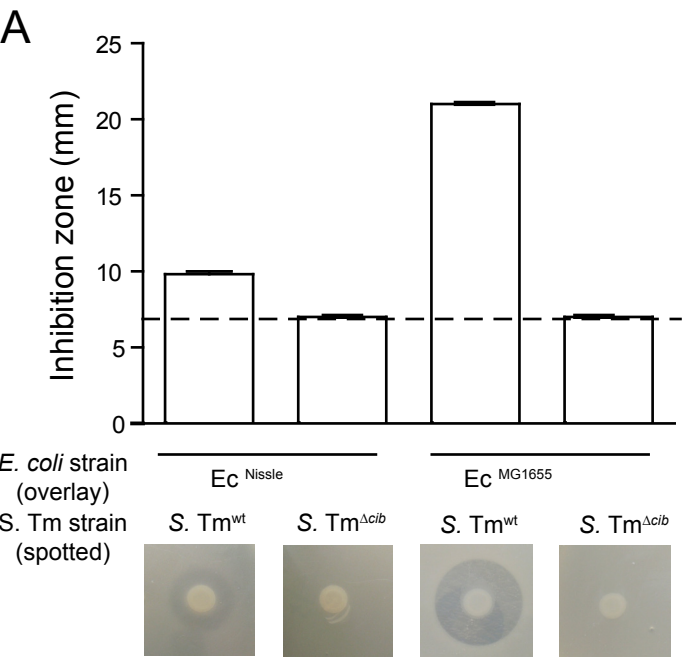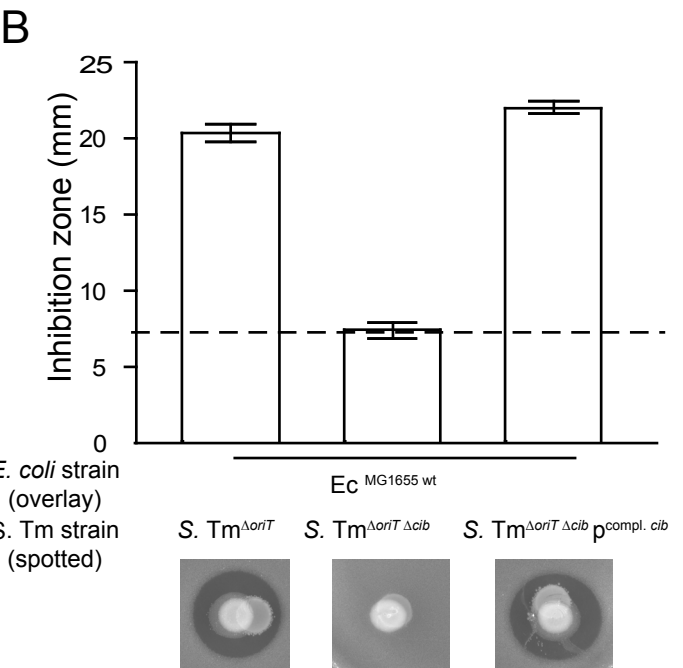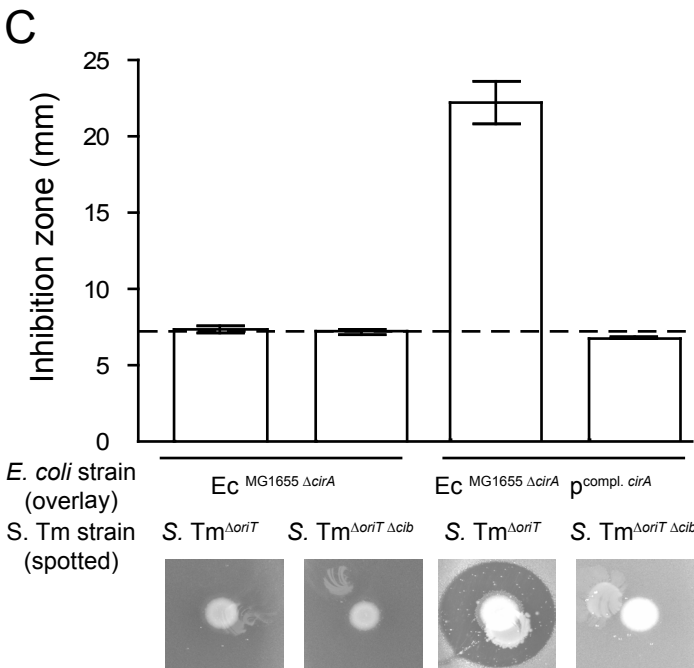

Supplement: Figure S1 — Halo-assay to confirm phenotypes of ColIb production and susceptibility. (A) ColIb susceptibility of EcNissle and EcMG1655. S. Tmwt was spotted on LB agar plates containing mitomycin C and incubated o.n. to induce ColIb secretion. EcNissle and EcMG1655 were cultivated in LB medium o.n., mixed with soft agar and overlaid on the agar plates. (B) Plasmid-based complementation of the ColIb-deficient S. Tm mutant S. TmΔoriT Δcib. S. TmΔoriT, S. TmΔoriT Δcib and S. TmΔoriT Δcib pcompl. cib were spotted on LB agar plates containing mitomycin C and incubated o.n. to induce ColIb secretion. EcMG1655 was cultivated in LB medium o.n., mixed with soft agar and overlaid on the agar plates. (C) Plasmid-based complementation of the CirA-deficient EcMG1655 ΔcirA mutant. S. TmΔoriT, and S. TmΔoriT Δcib were spotted on LB agar plates containing mitomycin C and incubated o.n. to induce ColIb secretion. EcMG1655 ΔcirA and EcMG1655 ΔcirA pcompl. cirA were cultivated in LB medium o.n., mixed with soft agar and overlaid on the agar plates. The experiments were done in triplicates and the diameter of the ColIb inhibition zone (halo) was measured after 24 hours. The detection limit (dotted line) is the average size of the S. Tmwt colony. (PDF) [file ppat.1003844.s001.pdf]

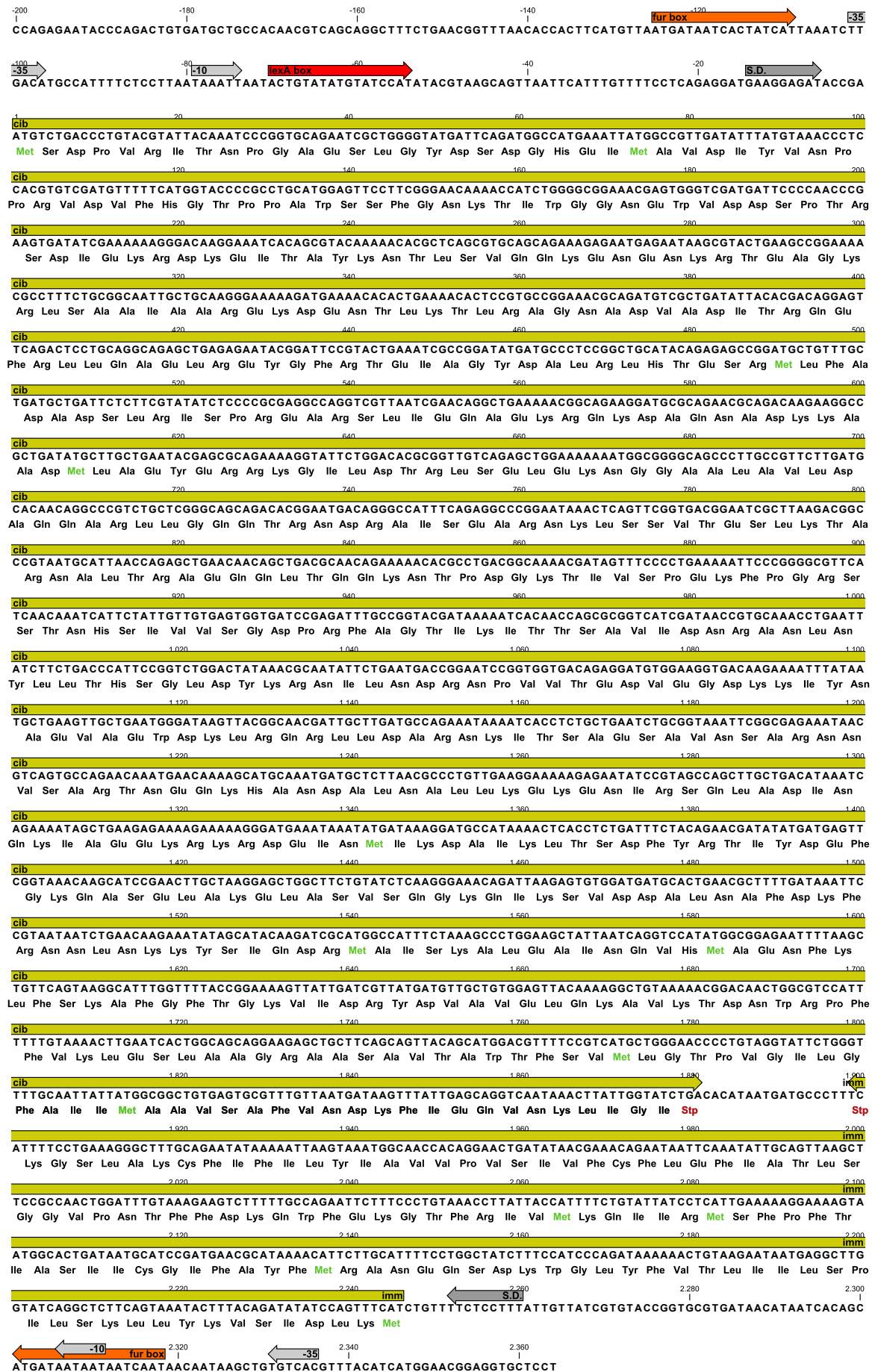

Supplement: Figure S2 — Nucleotide sequence of S. Tmwt cib imm and its respective promoter regions. Fur- and LexA repressor binding sites were annotated to the cib and imm sequence region of S. Tmwt as described in the materials and methods section. The position of the Fur-box, LexA-box, major transcription start sites and their corresponding −10 and −35 regions are indicated, as well as the open reading frame and the prospective ribosome-binding site (S.D.). (PDF) [file ppat.1003844.s002.pdf]

# Nedialkova, Denzler et al., Figure S3

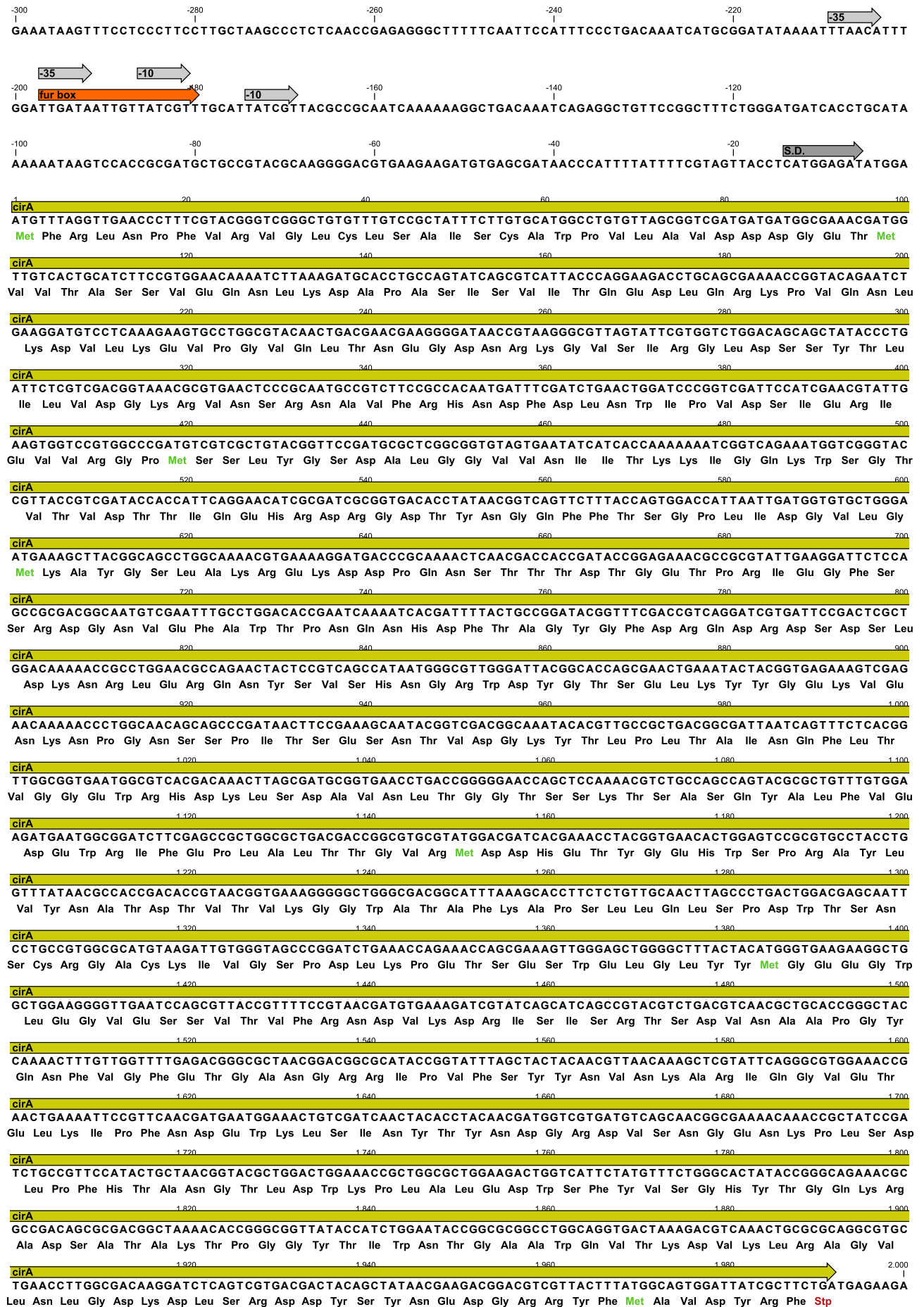

Supplement: Figure S3 — Nucleotide sequence of EcMG1655 cirA and its promoter region. Fur-repressor binding site was annotated to the cirA sequence region of EcMG1655 as described in the materials and methods section. The position of the Fur-box, major transcription start sites and their corresponding −10 and −35 regions are indicated, as well as the open reading frame and the prospective ribosome-binding site (S.D.). (PDF) [file ppat.1003844.s003.pdf]

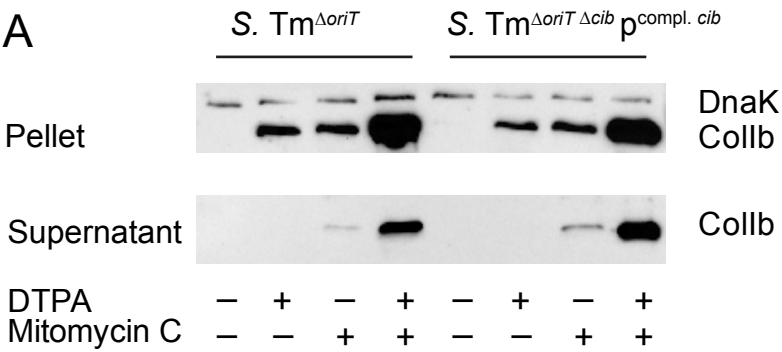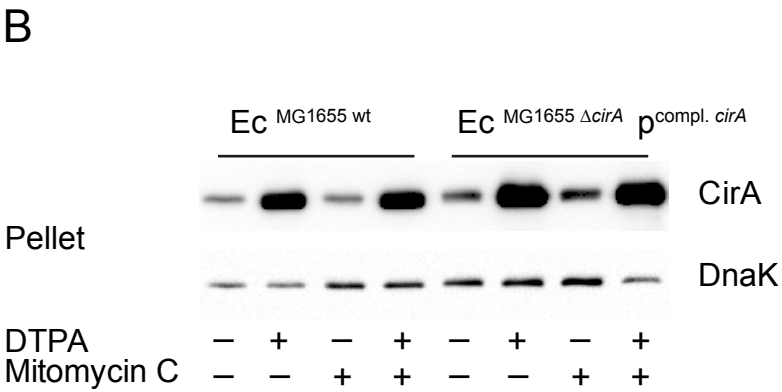

Supplement: Figure S4 — >Characterization of plasmid-based complementation of S. TmΔ oriT Δcib and EcMG1655 Δ cirA mutant strains by western blot. Overnight cultures of indicated S. Tm (A) and EcMG1655 strains (B) were re-inoculated 1∶20 in fresh LB with indicated supplements (0.25 µg/ml mitomycin C; 100 mM DTPA) and grown under aeration for 4 h. Cultures were normalized to OD600, bacteria were harvested and ColIb was detected in bacterial lysates as well as in the culture supernatant by immunoblot using an affinity-purified rabbit-α-ColIb antiserum (A). S. Tm DnaK was detected as loading control (A). EcMG1655 CirA was detected in bacterial lysates by immunoblot using a rabbit-α-CirA antiserum (B). E. coli cytoplasmic protein DnaK was detected as loading control (B). (PDF) [file ppat.1003844.s004.pdf]

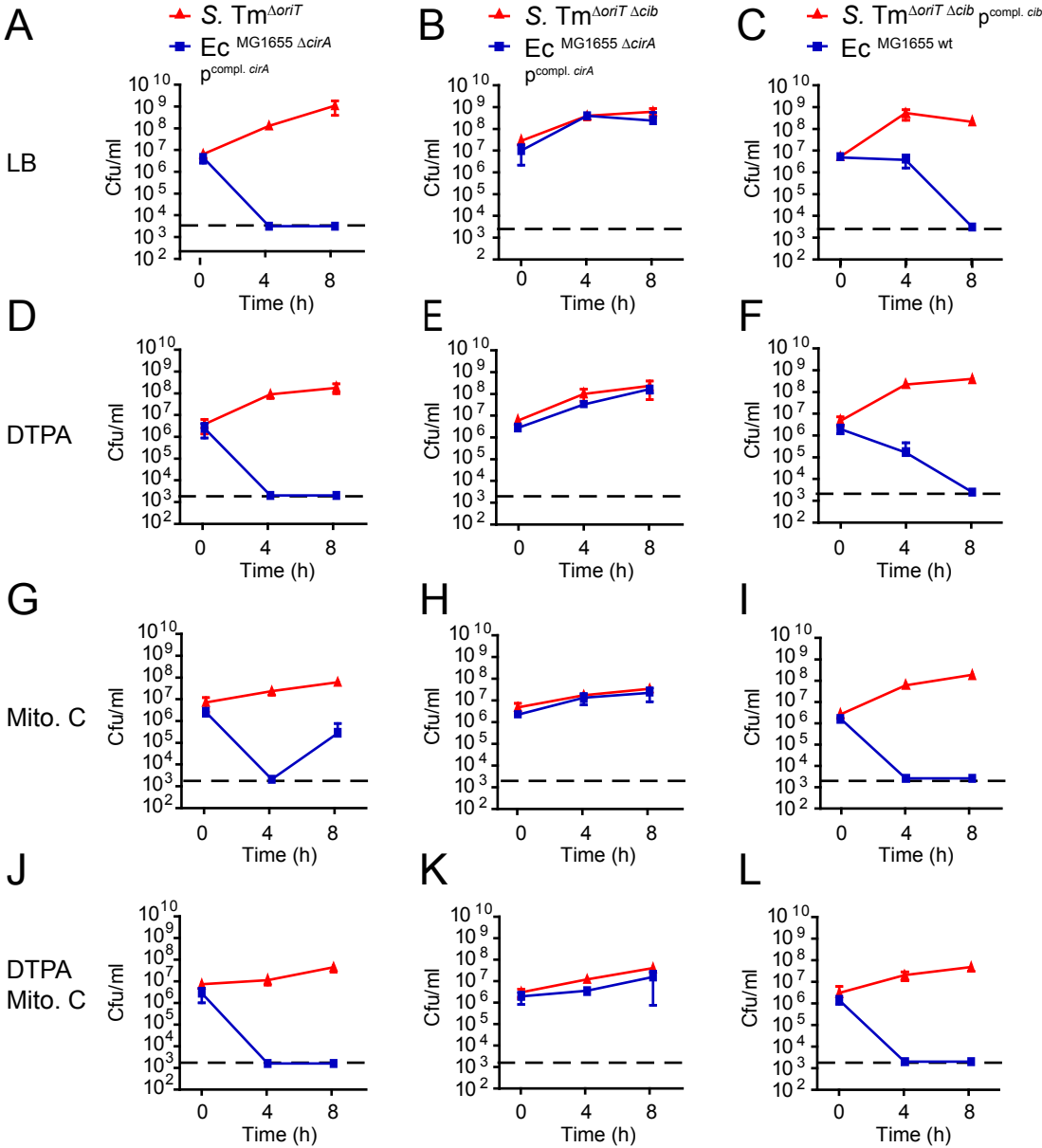

Supplement: Figure S5 — ColIb dependent competition of complemented S. Tm and E. coli mutant strains in vitro . Overnight cultures of S. TmΔoriT and EcMG1655 ΔcirA pcompl. cirA (A, D, G, J), S. TmΔoriT Δcib and EcMG1655 ΔcirA pcompl. cirA (B, E, H, K) or S. TmΔoriT Δcib pcompl. cib and EcMG1655 (C, F, I, L) were diluted and normalized to an OD600 of 0.05 for each strain in fresh LB with indicated supplements (0.25 µg/ml mitomycin C (Mito. C); 100 mM DTPA). Cfu/ml of both strains were determined at 0 h, 4 h, and 8 h after start of the subculture. Red lines: S. Tm strains, blue lines: E. coli strains. Dotted line: detection limit (2000 cfu/ml). Plasmid-based reconstitution of cib and cirA to the mutant strains leads to an over-complementation apparent by ColIb-dependent killing of E. coli in LB in the absence of supplements which is attributed to the multi-copy nature of the complementation-plasmid (A, C). (PDF) [file ppat.1003844.s005.pdf]
