# Supplementary material for: Inflammation Fuels Colicin Ib-Dependent Competition of Salmonella Serovar Typhimurium and E. coli in Enterobacterial Blooms
Source: PLoS Pathog. 2014 Jan 2;10(1):e1003844. doi: 10.1371/journal.ppat.1003844 (PMC3879352; doi:10.1371/journal.ppat.1003844)
Supplement: Table S1 — Primers used in this study. All PCR primer sequences used in the study are listed. (DOCX) [file ppat.1003844.s006.docx]

**Table S1. Primers used in this study**

| **Designation** | **Sequence** | **Used for** |
| --- | --- | --- |
| K12Δ*cirA*_rev | GCAGTATTTACTGAAGTGAAAGTCCGCCCGGTTCGCCGGGCATCTTCTCAtgtgtaggctggagctgcttc | LPN2 |
| K12Δ*cirA*_fwd | TGTTCCGGCTTTCTGGGATGATCACCTGCATAAAAAATAAGTCCACCGCGatatgaatatcctccttagtt | LPN2 |
| *cirA* - up | TTCCGGCTTTCTGGGATGATCAC | LPN2 |
| *cirA* - down | GCGTATTCAGCCGGGATATGATCAC | LPN2 |
| *cirA* - d1 | AGATCCGGGCTACCCACAATCTTAC | LPN2 |
| *ΔoriTnikA_*rev-val | GAAGCCATTGGCACTTTCTC | LPN5 |
| *ΔoriTnikA* val | AGTTCCTCATCGGTCATGTC | LPN5 |
| *ssaV*-check_fwd | GGAGCTCTGGTTACGATT | LPN5 |
| *ssaV*-check_rev | ATATTTCAGCCTCAGACG | LPN5 |
| For_colicin_*Nhe*I | CCCGCTAGCATGTCTGACCCTGTACGTATT | pLPN14 |
| Re_colicin_*Xho*I | CCCCTCGAGGATACCAATAAGTTTATTG | pLPN14 |
| For_*cirA*_*Nhe*I | CCCGCTAGCATGCTGCCGTACGCAAGGGGA | pLPN13 |
| Re_*cirA*_*Xho*I | CCCCTCGAGGAAGCGATAATCCACTGC | pLPN13 |
| p*CirA*-*Bam*HI | CCCGGATCCCGCGGTGGACTTATTTGTATG | pLPN1 |
| p*CirA*-*Xba*l | CCCTCTAGATTCCTCCCTTCCTTGCTAAGC | pLPN1 |
| pColIb-*Xba*I | CCCTCTAGACGTCAGCAGGCTTTCTGA | pM1437 |
| pColIb-*Bam*HI | CCCGGATCCTCGGTATCTCCTTCATCC | pM1437 |
| Luc-for-*Bam*HI | CCCGGATCCTAAGAAGGAGATATACCATGG A | pLPN15, LPN16 |
| Luc-rev-*Hind*III | CGAAAGCTTACAATTTGGACTTTCCGC | pLPN15, LPN16 |
| pWSK29-Gbs-for | TAATTGCGCGCTTGGCGTAATC | pWRG693-1; pWRG694 |
| pWSK29-Gbs-rev | ACCCAATTCGCCCTATAGTG | pWRG693-1; pWRG694 |
| CirA-pWSK29-Gbs-for | CGTAATACGACTCACTATAGGGCGAATTGGGTCCTTGCTAAGCCCTCTCAAC | pWRG693-1 |
| CirA-pWSK29-Gbs-rev | CTATGACCATGATTACGCCAAGCGCGCAATTAGCGTATTCAGCCGGGATATG | pWRG693-1 |
| Cib-Imm-pWSK29-Gbs-for | CGTAATACGACTCACTATAGGGCGAATTGGGTCACCGAAATGGCTGGATAAC | pWRG694 |
| Cib-Imm-pWSK29-Gbs-rev | CTATGACCATGATTACGCCAAGCGCGCAATTAATACCCAGCCGCCAGAGAATAC | pWRG694 |
